# Biology-inspired data-driven quality control for scientific discovery in single-cell transcriptomics

**DOI:** 10.1186/s13059-022-02820-w

**Published:** 2022-12-27

**Authors:** Ayshwarya Subramanian, Mikhail Alperovich, Yiming Yang, Bo Li

**Affiliations:** 1 Klarman Cell Observatory, Broad Institute of MIT and Harvard, Cambridge, MA USA; 2 Brigham and Womens’s Hospital, Harvard Medical School, Boston, USA; 3 MIT PRIMES, Massachusetts Institute of Technology, Cambridge, MA USA; 4 Lexington High School, Lexington, MA USA; 5 Present Address: Wake Technical Community College, Raleigh, USA; 6 Center for Immunology and Inflammatory Diseases, Department of Medicine, Massachusetts General Hospital, Boston, MA 02114 USA; 7 Present Address: Department of Cellular and Tissue Genomics, Genentech Inc., South San Francisco, CA USA; 8 Department of Medicine, Harvard Medical School, Boston, MA 02115 USA

**Keywords:** scRNA-seq, Quality control (QC), Data-driven, Single cell, Adaptive QC, Exploratory data analysis (EDA), Biological variation

## Abstract

**Background:**

Quality control (QC) of cells, a critical first step in single-cell RNA sequencing data analysis, has largely relied on arbitrarily fixed data-agnostic thresholds applied to QC metrics such as gene complexity and fraction of reads mapping to mitochondrial genes. The few existing data-driven approaches perform QC at the level of samples or studies without accounting for biological variation.

**Results:**

We first demonstrate that QC metrics vary with both tissue and cell types across technologies, study conditions, and species. We then propose data-driven QC (*ddqc*), an unsupervised adaptive QC framework to perform flexible and data-driven QC at the level of cell types while retaining critical biological insights and improved power for downstream analysis. *ddqc* applies an adaptive threshold based on the median absolute deviation on four QC metrics (gene and UMI complexity, fraction of reads mapping to mitochondrial and ribosomal genes). *ddqc* retains over a third more cells when compared to conventional data-agnostic QC filters. Finally, we show that *ddqc* recovers biologically meaningful trends in gradation of gene complexity among cell types that can help answer questions of biological interest such as which cell types express the least and most number of transcripts overall, and ribosomal transcripts specifically.

**Conclusions:**

ddqc retains cell types such as metabolically active parenchymal cells and specialized cells such as neutrophils which are often lost by conventional QC. Taken together, our work proposes a revised paradigm to quality filtering best practices—iterative QC, providing a data-driven QC framework compatible with observed biological diversity.

**Supplementary Information:**

The online version contains supplementary material available at 10.1186/s13059-022-02820-w.

## Background

Single-cell RNA sequencing (scRNA-seq) offers unprecedented resolution into cell biology by characterizing the individual cells within a biological sample of interest. Quality control (QC) of the cells is a critical first step in any scRNA-seq data analysis, which typically takes place after alignment of the sequencing reads to the reference genome (or transcriptome), and generation of the cell-by-gene matrix of gene expression counts. The goal of such “cell QC” is to remove “poor-quality” cells, based on QC metrics such as the number of genes detected (“gene complexity” or “transcriptional diversity”), the number of unique molecular identifiers (UMIs) recovered (typical for droplet-based technologies), and the fraction of mitochondrial and ribosomal protein genes [1]. The guiding motivation is that tissue dissociation techniques stress the cells and as cells die, transcription tapers off, cytoplasmic transcripts are degraded, and mitochondrial transcripts dominate [2]. Thus, low complexity of genes and high mitochondrial read content have been used as a proxy for identifying poor-quality cells (or droplets with ambient RNA). As a corollary, high gene complexity has been used as a proxy for doublets or multiplets in droplet-based sequencing [3]. While specialized computational strategies have been developed for other specific QC tasks such as ambient RNA correction [4–6], empty droplet removal [7], or doublet identification [8–10], the standard practice in “cell QC” is to filter out cells by setting arbitrarily defined thresholds on the QC metrics. Widely used pipelines [11, 12] by default set a flat filter on the QC criteria for each sample or sets of samples analyzed, agnostic of the dataset and biology under study.

Although widely used, data-agnostic QC filters do not account for the fact that variation in the commonly used QC metrics may also be driven by biology (in addition to technical factors). For example, mitochondrial transcript abundance is dependent on cellular physiology [13], and metabolically active tissues (e.g., muscle, kidney) have higher mitochondrial transcript content [14, 15]. Ribosomal protein gene expression has also been shown to vary by tissue [16] in human adults and mice [17]. Although biological variability in ribosomal protein gene expression has been reported [18], ribosomal protein gene expression is often conflated with technical artifacts or housekeeping transcription activity during analysis. Within each tissue, compartments and cell types may show further variability in these QC attributes. For example, the total number of genes expressed (gene complexity) varies with both cell type (cells with biologically distinct functions) and cell state (distinct physiological functions adopted by the same cell type) as seen during stages of mouse and human development [19]. Expression profiles also vary with progression through the cell cycle [20] or changes in cell volume [21]. Further, specific biological conditions or perturbations can lead to differences in these QC measures. For example, naive poised T cells are known to have higher ribosomal content [22, 23], as are malignant cells [24]. Activated lymphocytes such as innate lymphoid cells (ILCs) [25] have greater transcriptional diversity, in an activation and condition-dependent manner. Thus, the commonly used QC metrics can exhibit widespread biological variability bringing to the center the biological context of the study.

The importance of calibrating cell QC for the mitochondrial read fraction based on the mouse or human tissue of origin has been highlighted [26]; however, the proposed upper limit of 5 or 10% was largely based on existing data at the time of the study. Newer technologies (e.g., 10x v3 chemistry) may need a variable cutoff for mitochondrial read fraction [27]. The *scater* package [28] encourages the use of diagnostic plots and sample-specific QC. More recently, probabilistic mixture modeling has been favored for data-driven quality control at the level of samples or sample sets, either in combination with other QC approaches [15] or standalone as in miQC [29]. However, no approach performs quality control explicitly considering the biological variability of QC metrics at the cell type or cell-state level.

Here, we survey the variability of QC metrics across diverse scRNA-seq datasets at the tissue and celltype level, demonstrate the need for a data-driven quality control approach that accounts for the biological variability of QC metrics at the level of cell types, and present a framework for data-driven QC (*ddqc)*, inspired by unsupervised approaches in single-cell analysis, that performs adaptive quality control while retaining biological insights. *ddqc* partitions data by filtering out cells that fail adaptive thresholds on QC metrics as determined by the median absolute deviation (MAD) on each cluster of cells. Finally, we demonstrate that *ddqc* retains cell types that are lost by conventional QC, expanding existing cellular taxonomies for tissues, and offering an opportunity for further exploration and biological discovery.

## Results

### Survey of QC practices suggests a need for data-driven QC

To study existing QC practices in cell filtering, we sampled 107 research papers (“Methods”) with publication dates between 2017 and 2020, and focusing on analysis of scRNA-seq data generated across a range of technologies (3’ 10x V2 and 3’ 10x V3, Smartseq2, Drop-seq, mCEL-Seq2, Dronc-seq, MIRALCS, Microwell-seq) and in two species (mouse and human [30]), and summarized the QC practices adopted (Additional file 1: Table S1). The most commonly used QC metrics were the number of genes detected, the number of UMIs counted, and the fraction of reads mapping to mitochondrial or ribosomal protein genes. While there were few studies that used study-specific QC thresholds (Additional file 2: Supplementary Text), most studies (Table 1) that applied cell QC on specific metrics used data-agnostic QC filters, usually set at 5–10% for fraction of mitochondrial reads (86% or 73 papers), and 500 for gene complexity (86.5% or 77 papers).

**Table 1:** Summary of QC survey

Metric\QC type	Papers with any QC	Data-agnostic fixed threshold (% of filtered)	Multiple fixed thresholds varying by sample	Mito or ribo genes removed before analysis	Data-driven study-level threshold	Custom QC	No filtering
nCounts	65	48 (73.8%)	5 (7.7%)	0 (0%)	11 (16.9%)	1 (1.5%)	42
nGenes	89	72 (80.8%)	5 (5.6%)	0 (0%)	12 (13.5%)	0 (0%)	18
nCells	41	35 (85.4%)	3 (7.3%)	0 (0%)	2 (4.9%)	1 (2.4%)	65
%Mito	85	69 (81.2%)	5 (5.9%)	4 (4.7%)	6 (7.1%)	1 (1.2%)	22
%Ribo	7	2 (28.6%)	0 (0%)	3 (42.8%)	2 (28.6%)	0 (0%)	100
Empty droplets			Doublets/multiplets		Ambient RNA		
4			17		6		

### Across species and technologies, QC metrics vary by tissue

To systematically investigate if scRNA-seq data generated by commonly used technologies retains tissue and celltype specificity of the QC metrics, we profiled QC statistics by tissue and cell type on large public datasets after minimal basic QC (“Methods”). We surveyed 5,261,652 cells from 498 samples and 47 human tissues across 34 studies [31–54], and 966,560 cells from 337 samples and 37 mouse tissues across 5 studies [55] (“Methods”, Additional file 1: Table S2). We examined 8 human tumor types across protocols (fresh cells/scRNA-seq vs frozen nuclei/snRNA-seq) and droplet chemistries (10x v2 vs 10x v3) [27]. A subset of the studies (*Tabula Muris* [56] 10X, *Tabula Muris* Smartseq2; Microwell-seq mouse [57] and human [42]; *Tabula Muris Senis* [58]) had both uniformly generated and processed datasets, while others (PanglaoDB [59–67]) were generated in independent studies but uniformly processed. The mouse *Tabula Muris* dataset was particularly convenient having data generated from both 3’-end droplet-based sequencing (10X, (Additional file 3: Fig. S1A, C, E)) and full-length RNA plate-based Smartseq2 techniques (Additional file 3: Fig. S1B, D, F) from the same samples, and processed uniformly using the same reference and computational pipelines.

**Fig. 1:**
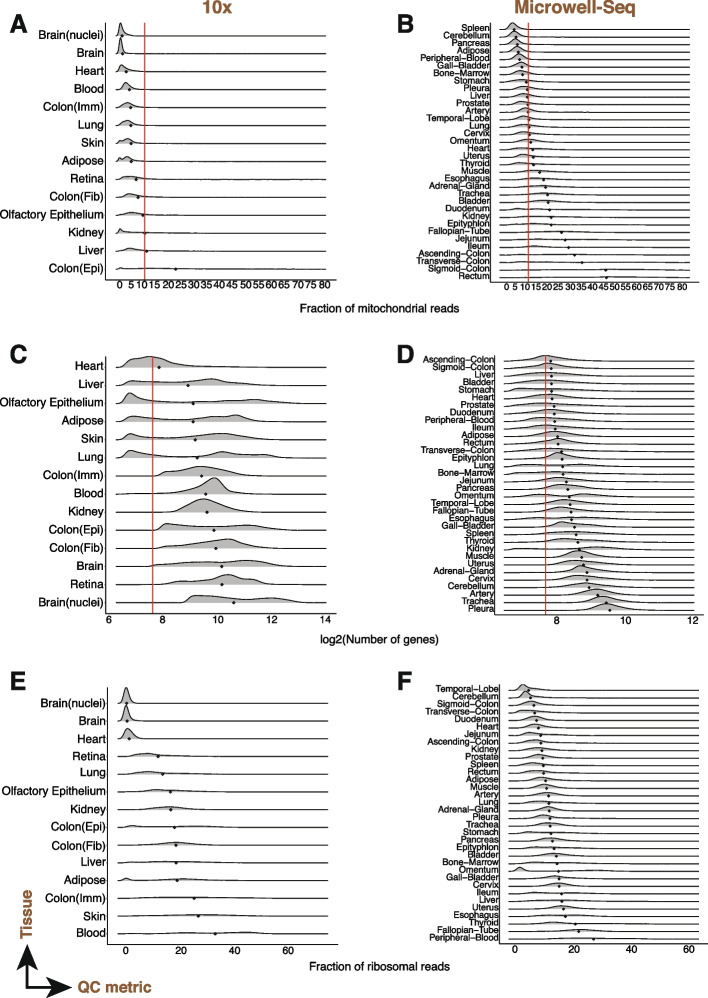
QC metrics vary by tissue. (*X*-axis) Fraction of mitochondrial reads (**A, B**), gene complexity (**C, D**), and percentage of ribosomal protein genes (**E, F**) per cell across human tissues (*Y*-axis) and technologies. Various human tissue scRNA-seq datasets generated by 10X droplet-based (**A, C, E**) and Microwell-seq (**B, D, F**) technologies. Each row in a panel is a density curve with the mean represented by a blue diamond. Red lines indicate conventional threshold values set at 10% for percentage of mitochondrial reads, and 200 for gene complexity

We found a tissue-specific (Fig. 1) trend for the QC metrics across studies. In general, we found variation by tissue for proportion of mitochondrial reads (Fig. 1A, B) within the same study regardless of the technology used (*Tabula Muris* 10X, *Tabula Muris* Smartseq2; Microwell-seq mouse and human) with some tissues emerging as having higher mitochondrial content (e.g., kidney, colon, heart, liver). The tissue-specific ordering of mitochondrial reads seen in [13] was most faithfully recapitulated by the Smartseq2 dataset (Additional file 3: Fig. S1B) with kidney, colon, cerebellum, and heart having the highest mitochondrial load. Differences in the gene complexity (Fig. 1C, D) and the percent of ribosomal protein genes (Fig. 1E, F) were also observed among tissues. Across both *Tabula Muris* 10X and *Tabula Muris* Smartseq2, the tongue had the highest mean gene complexity (Additional file 3: Fig. S1C, D), with the mean percentage of ribosomal protein reads being higher in the 10X dataset (Fig 1E). Trends were generally also maintained with age (*Tabula Muris Senis* 30m, Additional file 3: Fig. S2A, C, E). When compared to frozen tumor nuclei, the gene complexity was higher for cells (Additional file 3: Fig. S2D). Further, within each tissue, multiple density modes were evident (Fig. 1) for the QC metric studied. Finally, we note that the summary statistics of the QC metrics can vary by the experimental condition (technology and study) on the same tissue.

### Across species and technologies, QC metrics vary by cell type within a tissue

**Fig. 2:**
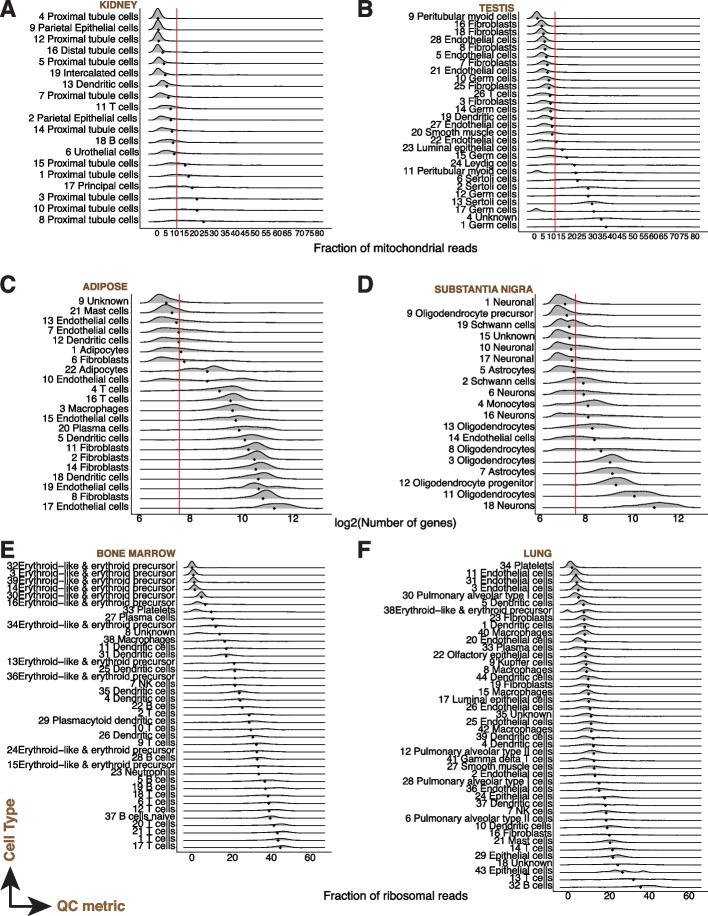
QC metrics vary by celltype. (*X*-axis) Fraction of mitochondrial reads (**A, B**), gene complexity (**C, D**), and percentage of ribosomal protein genes (**E, F**) per cell across cell types (*Y*-axis) of various human tissues: kidney (**A**), testis (**B**), adipose (**C**), substantia nigra (**D**), bone marrow (**E**), and lung (**F**). All scRNA-seq data was generated using the 10X droplet-based technology. Each row in a panel is a density curve with the mean represented by a blue diamond. Red lines indicate conventional threshold values set at 10% for percentage of mitochondrial reads, and 200 for gene complexity. Cluster numbers are indicated preceding the cell type annotation

We next assessed cell subset-specific (representing cell types or cell states) QC attribute differences within tissues by uniformly processing all datasets (starting with the gene expression count matrices) to derive clusters within each tissue without applying standard QC cutoffs (“Methods”). However, many publicly available datasets did not come with assigned celltype annotations. To uniformly assign biological annotations to the cell clusters, we devised a heuristic score function leveraging the top differentially expressed genes in a cluster, and the PanglaoDB [59] database of marker genes to predict the most probable cell-type annotation. We tested the annotation strategy on 4 mouse (*Tabula Muris* Smartseq2, *Tabula Muris* 10X, *Tabula Muris Senis* 24 months, *Tabula Muris Senis* 30 months) and 1 human (Human Tissue Atlas) datasets which had partial annotations provided by the authors. On these data, our heuristic approach had an accuracy of 80.2 and 92.1% for cluster annotations in human and mouse data respectively (Additional file 1: Table S3, “Methods”). We applied our heuristic approach to all test datasets and then examined trends of the QC metrics among cell types within tissues. As case studies, we manually verified annotations and describe examples for murine (Additional file 3: Fig. S3) and human tissues (Fig. 2).

Across all tissues, we observed variability by annotated cell type, in the per cell QC metrics (fraction of mitochondrial and ribosomal reads mapped, and gene complexity per cell). To illustrate the impact of standard practice QC thresholds, we applied QC thresholds of 10% for the maximum mitochondrial read fraction and 500 genes detected for minimum gene complexity. A fixed cutoff of 10% mitochondrial read fraction led to loss of parenchymal cell subsets in human kidney and testis (Fig. 2A, B), and mouse cerebellum, and colon (Additional file 3: Fig. S3A,B). More broadly, mitochondrial-read-rich clusters ranged from muscle cells to tissue-parenchymal cells such as enterocytes (gut), proximal tubular cells (kidney), or sertoli cells (testis), all cell types known to have high metabolic activity and energy needs such as active transport in the kidney proximal tubule, and oxidative phosphorylation in cardiomyocytes of the heart. Even a conservative fixed cutoff of 200 genes led to loss of diverse cell subsets including immune cells such as neutrophils (Additional file 3: Fig. S3C, D) and neurons (Fig. 2D). Cell type-specific trends in percent ribosomal protein genes were also evident (Fig. 2E, F, Additional file 3: Fig. S3E,F). Thus, data-agnostic thresholds remove biologically relevant cells, and hence, QC based on these metrics must not only adapt to different tissues or samples but also to cell states and cell types.

### *ddqc*: a cell-state adaptive quality control framework

**Fig. 3:**
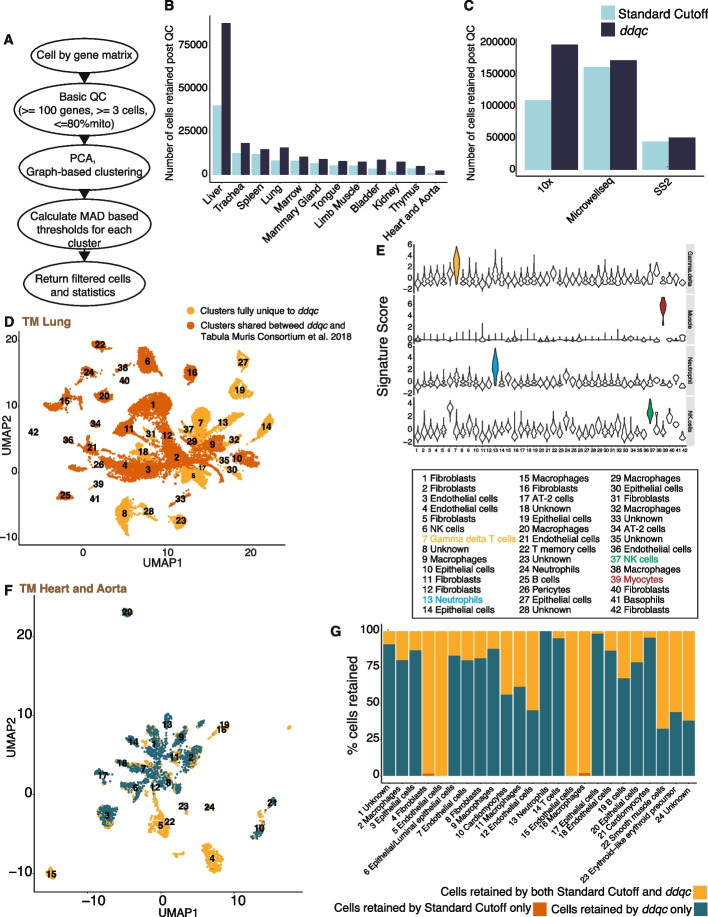
*ddqc* retains biologically meaningful cells that conventional QC filters out. **A** Overview of the *ddqc* approach. **B, C** *ddqc* retains more cells when compared to the standard cutoff approach across **B** tissues in the *Tabula Muris* dataset, and **C** scRNA-seq data generating technologies. **D** UMAP visualization of *Tabula Muris* lung cells. Colors represent whether the cells are included in the paper or uniquely retained by *ddqc*. **E** Violin plot visualization of cell type-specific signature scores in average log(TPX+1). From top to bottom: muscle, neutrophil, NK cells, and Gamma-delta T cells. **F** UMAP visualization of joint clustering of cells retained by both *ddqc* and the standard cutoff in the *Tabula Muris *heart and aorta tissues. **G** Proportion of cells retained by *ddqc*, standard cutoff, or both in the mouse heart and aorta tissues

To account for biological variability among QC metrics, and also adapt to differences in experimental conditions (study design, technology, etc.), we propose data-driven QC (*ddqc*, Fig. 3A), an unsupervised, data-driven, and adaptive thresholding framework for optimal capture of biological diversity. Inspired by and adapting existing unsupervised approaches in scRNA-seq analysis [68], *ddqc* identifies neighborhoods of cells by graph-based clustering and performs QC on these clusters using an adaptive thresholding approach. The basic concept is that data must be partitioned by biology and that QC must be performed on these independent partitions. Briefly, cells that pass empty droplet filters are subjected to dimensionality reduction by principal component analysis, followed by nearest neighbor graph construction and clustering to identify cell clusters with similar transcriptional states (details in “Methods”). Our approach does not rely on prior annotation, rather it identifies biologically similar cells based on the density of the transcriptional data. Within each such cluster, we identify “outliers” based on one- or two-sided thresholds on the QC metric of interest, defined as those cells that lie beyond a chosen number of median absolute deviations (MAD) from the cluster QC metric distribution median. Cells that pass these thresholds then enter downstream analysis.

The specific downstream analysis depends on the study and biological questions of interest. For example, the next step may range from integration with other data modalities (e.g., spatial data) or batch effect correction or cell classification. If the next step is indeed conventional analysis involving clustering-based cell-type/state identification, followed by differential gene expression, analysts may choose to start with the clustering labels that *ddqc* generates during QC (and returns as an output) to merge, re-cluster, or subcluster based on their research question. *ddqc* is available as both R and Python packages on GitHub and can be readily plugged into standard scRNA-seq analysis pipelines such as Pegasus [69] or Seurat [12]. Flexible options and exploratory plots are provided to the user for more control. *ddqc* is agnostic to the approach used to remove empty droplets (Additional file 2: Supplementary Text) which can be user defined. Our results were robust to varying clustering algorithms (Additional file 3: Fig. S4A, B, Additional file 1: Table S4) or hyperparameters at the different steps (Fig S4C,D). Clustering approaches perform on par with automated cell-type annotation methods [70–72] (Additional file 1: Table S5). To help evaluate the MAD multiplier parameter to use as the adaptive threshold, *ddqc* provides exploratory plots. Our extensive evaluation supports an analyst involved interactive analysis that integrates EDA and the analyst’s expertise in the problem of interest.

We evaluated the performance of *ddqc* on all test datasets (Additional file 1: Table S2) applying adaptive QC on three QC metrics: fraction of UMIs mapped to mitochondrial genes, gene complexity, and number of UMIs. For comparisons, we ran conventional QC (“standard cutoff”) on our test datasets using a fixed threshold of 10% as the maximum fraction of mitochondrial reads, and 200 as the minimum gene complexity. We then evaluated the cells that passed QC by either approach in a number of ways: ability to (1) improve power, (2) expand existing cellular taxonomies, (3) recover biologically meaningful states, and (4) discover broadly useful insights of transcriptional activity. At instances, we use the terms cell “states” and “types” interchangeably as there may be multiple clusters with identical celltype markers, potentially representing biological states.

### *ddqc* improves power for downstream analysis when compared with conventional QC methods

We computed the number of cells retained by either *ddqc* or conventional QC and determined the breakdown by QC attributes. *ddqc* preserved more cells in comparison to conventional QC across datasets and biological conditions (Additional file 1: Table S6). Overall, *ddqc* retained up to a median of 95.4% of input cells versus 69.4% cells using the standard cutoff approach. The higher number of cells retained by *ddqc* held across tissues (Fig. 3B) and technologies (Fig. 3C). Stratified by QC attributes, on average 83.19% of cells lost by *ddqc* are due to thresholds on the proportion of mitochondrial reads while 6.2% are lost due to gene complexity (Additional file 1: Table S6) thresholds. Thus, the higher number of cells preserved by *ddqc* provides more statistical power for downstream analysis.

### *ddqc* retains biological cellstate information lost using default cutoff or data-driven approaches that do not consider biology

As *ddqc* applies QC per cluster, it helps retain several cell types or states of biological relevance. We illustrate the biological relevance of *ddqc* in two ways. First, using the *Tabula Muris* lung dataset as a case study, we compared changes in lung cell taxonomies derived by conventional clustering analysis following either *ddqc* or the author-defined cutoffs. In the *Tabula Muris *paper, the authors used fixed cutoffs of 500 genes for minimum gene complexity and 1000 UMIs for the minimum number of UMIs. After QC by *ddqc*, we overlaid cell barcode annotations (Fig. 3D) provided by the authors [56] to define clusters with cells retained both in the paper and *ddqc*, and those exclusively retained by *ddqc* (i.e., all cells in the cluster were filtered out in the paper but retained by *ddqc*). Examining clusters exclusively retained by *ddqc*, we find various cell types of interest such as muscle cells, neutrophils, Natural Killer (NK) cells, and T cells, which we validate using their known canonical signatures (Fig. 3E, Additional file 1: Table S7). These cell states were filtered out in the *Tabula Muris* study and not analyzed downstream. When these data are lost, we also lose the biology or insights we might have learned by analyzing them. Thus, using *ddqc*, we are able to expand tissue cellular taxonomies by retaining tissue-native cell types missed by arbitrary cutoff-based QC.

Next, to demonstrate that *ddqc* recovers biologically meaningful states, we proceeded to annotate the cells that passed QC using our heuristic annotation strategy. Since our annotation strategy labels cell clusters and not individual cells, we jointly clustered the cells retained by both *ddqc* and the standard cutoff QC, and then applied our heuristic clustering strategy to assign biologically relevant labels. To evaluate differences in the filtered cells by both approaches, we defined “uniquely retained” clusters as those that had at least 30 cell members, and 85% of cluster membership consisted of cells uniquely retained by either QC method. No cluster was unique to the standard cutoff approach by the above definitions whereas several biologically meaningful clusters were uniquely retained by *ddqc* (Additional file 1: Table S8). We describe three examples: *Tabula Muris* heart and aorta (Fig. 3F, G, Additional file 3: Fig. S4E, G), human Olfactory Epithelial cells (Additional file 3: Fig. S4F,H, S5A,B), the human lung (Fig S5C,D). Compared to the standard cutoff method, *ddqc* retained cell subsets with low gene complexity including olfactory epithelial cells, dendritic cells, erythroid precursor cells, and platelets which were filtered out by the conventional QC approach. Cardiomyocytes (Additional file 3: Fig. S3A) and lung muscle (Fig. 3G) cells were mito-rich and retained in *ddqc*. The majority of cells with high mitochondrial content are diverse epithelial cells in both mouse and human. We provide a table of cell states lost when conventional methods are used across all our surveyed datasets (Table S8).

Finally, to compare with a data-driven approach, we ran miQC using standard settings (“Methods”) on the human olfactory epithelium and the mouse heart datasets (Additional file 3: Fig. S5E, F). For the human Olfactory Epithelium, both *ddqc* and miQC retain all clusters (miQC retaining up to 95% of cells as ddqc) with *ddqc* retaining more of mito-rich olfactory epithelial cells. However, in the* Tabula Muris* mouse heart example (Additional file 3: Fig. S5E), miQC retained only 90.5% of cells as *ddqc*, completely removing the cardiomyocyte cluster. The cardiomyocyte cluster had a median of 15.178% reads mapping to mitochondrial genes, and 2427.67 as the median gene complexity, which *ddqc* retains. Cardiomyocytes are essential parenchymal cells of the heart. In both examples, miQC retained fewer cells exclusively (that *ddqc* did not); however, these did not map to a missing biologically relevant cell type. Thus, *ddqc* retains biologically relevant cell types that miQC filters out.

### Which cells have the least and most number of transcripts?

**Fig. 4:**
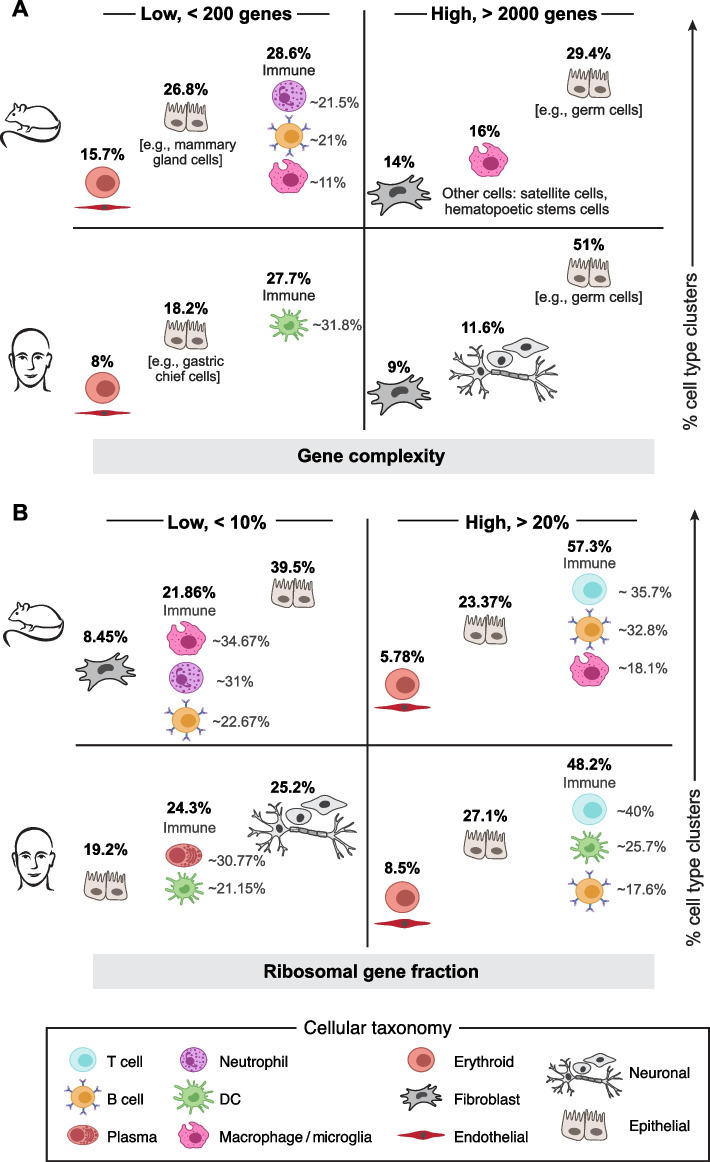
Schematic summarizing trends in “low” and “high” transcriptional diversity among mouse and human cell types for total number of genes (**A**) and ribosomal gene fraction (**B**). Top row represents the most prevalent cell type within the group (% of total clusters examined) for mouse and bottom row for human. Cell types are further partitioned within the immune cell category. **A** Gene complexity trends. Cell types with number of genes < 200 median number of genes detected are in the low gene complexity group while those with > 2000 median number of genes detected are in the high category. **B** Ribosomal gene fraction trends. Cell types with median fraction of ribosomal genes < 10% are in the low gene complexity group while those with median fraction of ribosomal genes > 20% are in the high category

We next turned to insights such as patterns of celltype-specific gene usage that a more biology-driven QC approach such as *ddqc* preserves (Fig. 4). Following application of *ddqc*, we examined trends in QC metrics (Additional file 1: Table S9), to answer questions such as “which cell types or states transcribe the fewest or largest number of genes?”. We defined cell states with low gene complexity as those with both low median number of genes detected (< 200) as well as low median percentage of mitochondrial reads (<10%). Across 20 human studies and 159 clusters, 44 of the clusters (27.7%) satisfying the criteria were diverse immune cells including dendritic cells, plasma cells, T cells, NK, and mast cells. Other subsets included endothelial subsets, platelets, and RBCs (6%). Specific parenchymal cells with low gene complexity were specialized cells such as gastric chief cells (*PGA5*^*+*^, *PGC*^*+*^, *CHIA*^*+*^, *PGA3*^*+*^, *LIPF*^*+*^) of the stomach, cardiomyocytes (*NPPA*^*+*^, *NACA*^*+*^, *NACA2*^*+*^, *MYL2*^*+*^), neuronal subsets (Schwann, astrocytes, neurons) of the substantia nigra, and olfactory epithelial cells. Across 4 large mouse studies and 465 clusters, 133 (28.6%) were immune cell clusters including 28 neutrophil (*Elane*^*+*^, *Prtn3*^*+*^, *Mpo*^*+*^) subsets, 27 B cells, and 46 macrophage/Kupffer subsets. Endothelial (46) and erythroid (23) lineages followed. Parenchymal cells included lactating and involuting mammary gland cells, pancreatic acinar cells, and diverse epithelial cells.

Next, we looked at cell types/states with high gene complexity (> 2000 median genes, < 10% fraction mitochondrial reads). Among 311 such clusters in humans, neurons (35), and fibroblast (28) emerged as the higher ranked ones, along with epithelial cells (159). In mice, across 377 clusters, macrophages/microglia (61), fibroblasts (53), neuronal (20), and diverse epithelial cells (111) were among the most populous subsets with high gene complexity.

### Immune cells have a high fraction of ribosomal protein content

Examining trends of ribosomal protein transcription, we defined high or low median ribosomal protein gene complexity as that with greater than 20% reads or lower than 10% reads mapping to ribosomal protein genes, and lower than 10% reads mapping to mitochondrial genes. Among 438 human clusters with high ribosomal protein gene complexity, 212 (48.4%) were immune cell subsets including 85 T cell, and 50 dendritic cell subsets. Immune cell function often requires rapid protein translation [23, 73]. Other preponderant subsets were epithelial (110) and fibroblasts (43). Among 450 such clusters in mice, 241(53.6%) were annotated as immune including diverse subsets (B cell (78), macrophages (44), and T cells (75)) suggesting that certain immune states may have high translational activity and need for ribosomal protein genes.

Neurons (20.6%) were a large fraction of human cell states with lower ribosomal protein gene complexity. In mouse, cell states with low ribosomal protein gene complexity included diverse epithelial and immune cells, fibroblasts, and endothelial cells. Thus, a more context-focused QC approach such as *ddqc* can enable us to recapitulate and study fundamental patterns in cell-type-specific gene expression and associated function.

## Discussion

Cell quality control remains an essential step in scRNA-seq data analysis; however, conventional approaches apply arbitrary filters on defined QC metrics without accounting for the biological context. The standard QC practice among published papers is largely data-agnostic and arbitrary threshold-based. We have demonstrated (Figs. 1 and 2) that not accounting for the underlying biological heterogeneity at the level of cell states during QC can lead to loss of relevant biological insights (including important cell types) as well as reduced statistical power for downstream analysis. However, identifying cell types and cell states is a time-consuming process requiring either well-annotated training sets or involves the manual and subjective task of cell-state annotation. The field of single-cell biology is still in the nascent stages of building experimentally validated and reproducible ontologies of cell states. The few existing automated annotation strategies are limited in the number of tissues they can handle. To overcome these challenges, we present an unsupervised approach *ddqc* that leverages clustering to identify transcriptionally similar cellular neighborhoods (approximating broad cell types) and performs adaptive QC on these clusters. The unsupervised approach underlying *ddqc* performs on par with independent annotation strategies on test datasets (Additional file 2: Supplementary Text).

We observe limitations of our approach: (1) *ddqc* applies adaptive thresholds on each cluster, and hence, we are likely to lose a small number of good-quality cells (false positives) due to inherent spread of the cluster data distribution. (2) While in most cases, *ddqc* retains clusters that are biologically meaningful, in some cases, *ddqc* may retain cells (Additional file 1: Table S8) with high percentages of mitochondrial genes that may be a mix of biology and technical artifacts. These clusters when sub-clustered do not always represent bimodal distributions (Additional file 3: Fig. S3), rather a gradation, and there is no perfect way to assess the right cutoff. Such cells are usually subsets of larger neighborhoods of biologically meaningful cells that reflect true metabolic stress due to the biological condition studied. In the current version of *ddqc*, removal of such cells has been left to the analyst after examination via Exploratory Data Analysis (EDA) in the context of the biology of the study, and during downstream analysis. We believe QC should be iterative and to help empower the user, *ddqc* provides detailed statistics for all cells that pass or fail adaptive QC.

*ddqc* provides several advantages relative to conventional cutoff or biology-agnostic data-driven approaches. First, it retains more cells than standard or data-driven QC approaches leading to more power for downstream analysis. Second, the additional cells retained by *ddqc* are biologically meaningful thus increasing the potential for further biological discovery. Such biological insights include retaining a diversity of cell types with extreme value QCs and rare cells, as well as uncovering study-specific metabolic and physiological programs that may dictate changes in these common QC metrics. Further investigation of retained cell states may provide insights into the underlying biological processes. Finally, we examine cells lost by conventional QC to add insights into questions of fundamental interest in biology such as parsimony in total gene usage or transcription. Our analysis has revealed interesting biological observations in terms of overall transcriptional diversity of cell states, as well as ribosomal protein gene expression.

## Conclusions

In summation, we propose a biology-centered and iterative approach to cell quality control for scRNA-seq data that retains cell states of critical biological relevance often removed by conventional QC. By contributing a framework for quality control that considers the biological properties of data, *ddqc* can revise how data analysis is performed in every scRNA-seq study.

## Methods

### QC survey

We conducted a survey of 107 single-cell and single-nucleus RNA sequencing papers published between 2017 and 2020. Papers included in the survey were collated either from Twitter posts, searches on Google, or a curated scRNA-seq database [74]. For each paper, we recorded the Quality Control (QC) strategy from the “Methods” section into Additional file 1: Table S1. Additional information was also recorded for each paper, including:

- Year published
- Organism
- Tissue of origin
- Sequencing technology
- Analysis software
- Preprocessing software

QC was classified into the following categories:

- QC to remove low-quality cells and genes by QC metric
  - ° Number of counts
  - ° Number of genes
  - ° Percent of mitochondrial transcripts
  - ° Percent of ribosomal transcripts
  - ° Number of cells in which gene is present
- QC to remove empty droplets
- QC to remove doublets/multiplets
- QC to account for ambient/background RNA

We categorized the papers based on the type of QC used for a particular metric. These categories were:

- Data-agnostic fixed threshold—QC removed all cells with a metric above/below a certain number (for example keep all cells with <10% mitochondrial transcripts)
- Multiple fixed thresholds—several fixed thresholds for different samples
- Data-driven study-level threshold—QC threshold was determined from the data (for example, keep all cells with a number of genes within 2 SDs from the median)
- Custom—QC that was very specific for the particular paper
- No filtering—no filtering based on this metric was done

The summary of the QC survey and QC methods are documented in Table 1 and the “Results” section.

### Datasets

We downloaded publicly available mouse (*n*=5) and 32 human (*n*=32) (Table S2) single-cell (scRNA-seq) or single-nucleus (snRNA-seq) RNA sequencing datasets. We restricted our study to droplet- (10X Genomics), Microwell-seq, and plate-based (SmartSeq2) technologies from various tissues.

We downloaded data at the level of gene counts after preprocessing (genomic reference alignment and gene-level quantification) but prior to any quality control (QC). However, many datasets in public repositories were already filtered using cutoffs or were aligned to reference genomes with missing genes. In some cases, we were able to contact study authors (e.g., Tabula Muris) and get the unfiltered expression matrices. Links to the unfiltered datasets used can be found in Additional file 1: Table S2. Our dataset search was agnostic to the computational preprocessing methodology or genome reference version used.

### Input files

For all analyses, we start with loading the unfiltered or raw cell-by-gene matrix stored either in the mtx, csv, txt, or h5ad format.

### *ddqc*

We propose an adaptive thresholding method to perform quality control at the level of cell types, thus taking into account differences between them. The first step of this method is to cluster the cells using standard scRNA-seq analysis preprocessing and clustering steps. We assume that within each cluster, cells are of the same or closely related cell type with shared biological properties. In each cluster, we expect outliers—cells with the number of UMI counts, number of genes, or percent of mitochondrial transcripts significantly different from the cluster average. We assume that those differ in quality from other cells in their cluster and remove them by calculating a cutoff for each cluster based on median absolute deviation and a user-defined parameter x. We chose the median absolute deviation (MAD) to be a more robust statistic to define outlier thresholds instead of the zscore which assumes normality, or IQR which is less permissive. If the cell has a value higher (percent.mito) or lower (n_counts, n_genes) than x MADs from the median in its cluster, this cell will be filtered out; all remaining cells will be sent for downstream analysis. If the cluster *ddqc* threshold was bigger than 200 n_genes, or lower than 10% mito, we would set it to 200 or 10 respectively.

*ddqc* uses preprocessing and clustering functions provided by the Pegasus (https://pegasus.readthedocs.io/) for the Python package: https://github.com/ayshwaryas/ddqc. An R package using functions in Seurat is also available: https://github.com/ayshwaryas/ddqc_R.

Our pipeline starts with a loading of the unfiltered cell-by-gene matrix stored either in mtx, csv, txt, or h5ad format. Below, we list the Python *Pegasus* functions with the Seurat R functions in parenthesis.

- Initial or Empty droplet Filtering: by default, a minimal filtering is conducted to remove obvious low-quality cells or empty droplets: cells with less than 100 genes or with more than 80% of mitochondrial transcripts were removed using the Pegasus functions *qc_metrics* and *filter_data* (*subset* in R). Users may choose to skip the step, provide their own filters for each QC metric or provide filtered input files after applying an empty droplet detection method of their choice. For all analysis and results, initial filtering was conducted to remove poor-quality cells: cells with less than 100 genes or with more than 80% of mitochondrial transcripts and genes present in less than 3 cells are removed. The Initial Filtering step is essential for computational efficiency as otherwise, we may have on the order of a million or more barcodes in case of droplet-based scRNA-seq.
- Normalization is performed using the function *NormalizeData* (*NormalizeData* in Seurat): normalize the feature expression measurements for each cell by the total expression, multiply by a scale factor (10,000), and log-transform the result to get log(TPX+1) values.
- We find the top 2000 highly variable genes using the function call *highly_variable_features* (*FindVariableFeatures* in Seurat)*.* We scale the expression matrix of highly variable genes: shift the expression of each gene so that the mean expression across cells is 0 and scale the expression of each gene so that the variance across cells is 1 (In Pegasus, scaling is part of *pca*, in Seurat *ScaleData*)
- Next, dimensionality reduction is performed using principal component analysis (PCA) using *pca* (*RunPCA*) with the number of principal components set at 50.
- Graph-based clustering of cells was performed by first building the *k*-nearest neighbor graph setting *k*=20 [75], and then the Louvain algorithm for clustering [76] or community detection with the resolution set at 1.4 using the functions *neighbors* (*FindNeighbors*) and *louvain* (*FindClusters*) functions.
- Then we iterate through each of QC metrics to determine the cutoff values:
  - ° First we create a true/false numpy array (vector in R) that would represent whether the cells have passed ddqc
  - ° For each cluster, we find lower (for n_counts and n_genes, otherwise set to negative infinity) and upper (percent mito, otherwise set to positive infinity) cutoff (median ± *x* × MAD). *x* is user defined with a default of 2. For number of genes: If lower cutoff is more than 200 genes, it would be set to 200 (by default)
    - For percent mito: if upper cutoff is less than 10R%, it would be set to 10 (by default)
  - ° Finally, if the cell is outside the bounds defined by cutoffs, it would be marked as false in the ddqc array
- We do an *AND* operation between all ddqc metric-specific arrays. Cells that are marked as true in this array have passed ddqc and are retained for downstream analysis

In the Pegasus and Seurat workflows, in addition to returning the filtered object, ddqc returns a pandas dataframe with the following information for each cell:

- True/false value that indicates whether the cell passed the ddqc
- Cluster number that was assigned to this cell in the initial clustering
- For each QC metric:
  - ° The metric itself
  - ° Lower cutoff (cluster median − 2 cluster MAD) for this metric for the cell’s cluster. If there is no cutoff, this field will be equal to None
  - ° Upper cutoff (cluster median + 2 cluster MAD) for this metric for the cell’s cluster. If there is no cutoff, this field will be equal to None
  - ° True/false value that indicates whether the cell passed the ddqc for the given metric

In addition, the *ddqc* workflow displays four plots for exploratory data analysis:

- Two boxplots: one shows the percent mito by cluster with a red line at 10% that indicates the standard fixed threshold for percent mito, and the other shows the log2 of the n_genes by cluster with a red line at 200 genes (7.64 in log2-scale) that indicates the most commonly used fixed threshold for number of genes.
- If the MAD was selected as the threshold calculation method and the MAD multiplier was set using the *threshold* parameter of the *ddqc_metrics* function only, ddqc will generate two facet plots that show how the number of cells that are filtered out changes depending on the threshold value. These plots will help you to pick a threshold parameter if you want to tune it.

### Automated cell-type annotation

We automated the task of mapping cell type annotations to clusters using the PanglaoDB cell-type gene expression signatures as the reference dataset. Using the PanglaoDB cell-type:marker mappings, cell-type labels were assigned for each cluster as follows:

1. We computed cluster-specific differentially expressed genes (DGE) by testing for genes differentially expressed in the cluster of interest vs all else. For the testing, we used the default differential expression test used in Seurat for the R version or Pegasus for the Python version.
2. We filtered the DEG to retain those genes with at least a log fold change of > 0.25, percent expressed in the cluster of interest > 25%, and *q*_value < 0.05.
3. We iterated through each cluster to assign cell-type scores as follows:
  - First, we iterated through the filtered DEG of the current cluster to check for matches in PanglaoDB.
    i. If there was an entry that the gene indicates for a particular cell type, the average log fold change of that gene was added to the score of the cell type.
    ii. Only cell-type annotations which included at least three such marker genes were retained
  - The cluster was assigned the cell-type annotation with the highest score. Otherwise, the cell type would be stated as Unknown.

We note that the accuracy of our method is contingent on the accuracy of markers in the PanglaoDB dataset which would get updated on a regular basis. The PanglaoDB markers database does not have enough genes for certain cell types, which causes them to be assigned to similar but not identical cell types (For example, macrophages which are antigen-presenting cells (APC) are often labeled as dendritic cells, another APC). For examples in Figs. 2 and 3, annotations were manually verified.

### Automated cell-type annotation accuracy assessment

In order to assess the accuracy of our cell-type annotations method, we have compared the results of automated annotations with the annotation provided by the publisher of the dataset, if such annotation was provided. Datasets where the authors provided annotations included the Human Tissue Atlas; human adipose (inhouse annotated), heart, and lung; *Tabula muris* (10x), *Tabula muris* (Smartseq-2), *Tabula senis* 10x 24 and 30 month. The accuracy was calculated using the steps below.

1. First, we annotate the clusters after just the default empty droplet filters. We do it by mapping the annotation that is the most frequent among the cells of the cluster. If most of the cells do not have an annotation, the cluster will be marked as “unknown”.
2. For accuracy analysis, we are only including the clusters that had an annotation (not “unknown”) and where at least 75% of cluster cells had that annotation.
3. For the comparison, we have established a number of pairs of annotations that we considered to be the same **(**Additional file 1: Table S3). Some of these pairs are just different in naming between predicted and author-provided annotations (example NK cells VS Natural Killer cells), and others were validated by marker genes to be more accurately defined using our strategy than the author-defined annotations (e.g., cluster 6 in our analysis of the *Tabula Muris* Smartseq2 kidney dataset were author annotated to be collecting duct cells when they highly expressed loop of Henle and distal tubular markers *Umod* and *Slc12a1*, and which was correctly predicted by our algorithm).
4. Then, we count the number of clusters with a mismatch between automated annotation and the annotation provided by the publisher. If the annotation pair is included in the table from step 3, it will not be counted as a mismatch. After that, we compute the accuracy percentage.

The tables of the same cell types, mismatches, exact numbers, and breakdown by the dataset are provided in Additional file 1: Table S3.

### Comparison of ddqc with author-provided annotations

We have compared *ddqc* with author-provided quality control in *Tabula muris* tissue (Fig. 3):

1. First, the author-provided annotations were downloaded from figshare (https://figshare.com/articles/dataset/Single-cell_RNA-seq_data_from_microfluidic_emulsion_v2_/5968960?file=13088039).
2. Then we calculated the percent of cells exclusive to *ddqc* in each cluster after ddqc filtering (Additional file 1: Table S6). It was calculated by taking the number of cells whose barcodes were not present in author annotations (which means they were not included by the author for final analysis) and dividing it by the total number of cells in the cluster.
3. To verify the automated annotation for clusters where the number of cells exclusive to *ddqc* was 100%, we have computed signature scores for each of the clusters (using the “pegasus.calc_signature_score” function) with cell-type markers (Fig. 3E). You can find the signature genes in the Additional file 1: Table S6.
4. We have also generated UMAP plots with cells colored based on percent exclusive of their cluster. We had 2 categories: fully exclusive to *ddqc* or shared with the paper (Fig. 3D)

### Comparison of ddqc to the standard cutoff method

We compared *ddqc* with the standard cutoff or static threshold method (default in most pipelines) as a control, and only empty droplet filtering for reference:

1. *ddqc* using the same steps as described in the *ddqc* section for loading the data and filtering.
2. Standard cutoff or static threshold (cells with number of genes less than 200 and mitochondrial transcripts percent higher than 10% are removed regardless of filtering)
3. No QC (done for reference)

First, we evaluated the retained cells in all the three approaches independently by graph-based clustering, followed by differential gene expression using *de_analysis* function and UMAP visualization using *umap* the function. Also, additional statistics were recorded for future analysis (Information about clusters and cells). Exploratory data analysis (EDA) was performed by generating summary plots including boxplots, joyplots, and colored UMAP plots.

Next, for comparisons, we performed joint clustering as follows:

1. After QC was performed, each barcode is assigned a label which indicates if it was filtered or retained by each method. Possible options are retained by both methods, retained by ddqc only, retained by cutoff only, neither (removed by both cutoff and ddqc)
2. Barcodes that were marked as “neither” were removed
3. All remaining barcodes were clustered (as above) and visualized using UMAP.
4. Both cluster and filter labels were used to color the UMAPs for exploratory data analysis. Barplots were also generated per cluster to visualize the distribution of each cluster by cell retained in each method.
5. DGE was performed on the clusters to assign cell identity and to identify cell types lost by single-threshold QC.

These plots helped to demonstrate differences between static threshold and *ddqc* by highlighting clusters of cells that were kept by one method but lost by another.

#### Unique clusters

To demonstrate differences between static threshold (“cutoff”) and *ddqc*, we determined how many meaningful “unique” clusters *ddqc* retained. A “unique” cluster was defined as a cluster with at least 30 cells, and with at least 85% of its cells retained only by *ddqc* but filtered out by cutoff method. The presence of unique clusters indicates that a population of very similar cells was almost entirely filtered by one method, thus suggesting that potentially some cell types were exclusive only to the other method. This helped to demonstrate the advantage of *ddqc* over a static threshold since it had many more unique clusters than the static threshold method had. More detailed examples are provided in the “Results” section.

### Comparisons with miQC

At the time of testing, miQC was installed in R from GitHub using the command “remotes::install_github("greenelab/miQC", build_vignettes = TRUE)”. miQC was run on the test datasets (*Tabula Muris* heart and aorta and human olfactory epithelium) using the standard steps as described in the vignette: https://github.com/greenelab/miQC/blob/main/vignettes/miQC.Rmd. Comparison was performed by examining the intersection of miQC retained barcodes with those retained by *ddqc*, leveraging the annotations in the *ddqc* results.

### Trends table (Additional file 1: Table S8)

We determined trends in QC metrics by iterating through all ddqc clusters in all tissues and recording the clusters which satisfy one of the following criteria to a corresponding table:

- Median number of genes lower than 200
- Median number of genes higher than 2000
- Median percent mito higher than 10
- Median percent ribo lower than 10
- Median percent ribo higher than 20

### Comparison of clustering algorithms

In order to assess the performance of *ddqc* with different clustering algorithms, we have used 4 algorithms provided by Pegasus (louvain, leiden, spectral louvain, and spectral leiden), and also implemented two additional algorithms: *k*-means and hierarchical clustering. For the algorithms within Pegasus, the clustering was performed using the function *pegasus.louvain*, *pegasus.leiden*, *pegasus.spectral_louvain*, and *pegasus.spectral_leiden* respectively. The *k*-means and hierarchical clustering methods were implemented using *sklearn.cluster.KMeans* and *sklearn.cluster.AgglomerativeClustering* respectively. In both algorithms, *sklearn.metrics.silhouette_score* was used to determine the number of clusters. All functions were used with default parameters.

We ran *ddqc* using all 6 of those clustering algorithms for both initial and final clustering on Tabula Muris heart and aorta, and lung tissues. Then, we calculated the number of cell barcodes that were retained by *ddqc* in the results of all six algorithms, as well as the number of barcodes in pairwise intersections between different algorithms to determine if any one algorithm disproportionately retained more barcodes than the others.

### Assessment of ddqc performance on the Seurat PBMC dataset

We ran *ddqc* on the PBMC dataset provided in the Seurat tutorial vignette (https://satijalab.org/seurat/articles/get_started.html). To get the clustering labels provided in the tutorial, we repeated the tutorial steps in R and recorded the results into a csv file, cluster labels from which were later used as cell annotations for the ddqc run on the same data.

After comparing barcodes, we have identified that *ddqc* retains more cells than the Seurat filter, and we have identified those cells and their celltypes. Also, we have looked into logs provided by the *ddqc* to establish the cause of those cells being filtered out by ddqc.

### Comparison of *ddqc* with independent cell annotation methods

In order to assess the effectiveness of the graph-based clustering used in *ddqc* in parsing out biological heterogeneity, we compared it with independent classification methods to rule out any bias associated with clustering. We have used the following supervised and unsupervised methods:

1. SingleR [71]: https://bioconductor.org/packages/release/bioc/html/SingleR.html
2. Azimuth [72]: https://azimuth.hubmapconsortium.org/
3. CellTypist [70]: https://www.celltypist.org/

We have performed a comparison on Seurat PBMC dataset using the steps below:

1. First, we annotated each cell from the PBMC dataset provided in the Seurat tutorial vignette (https://satijalab.org/seurat/articles/get_started.html), following the steps described in the respective method (SingleR, Azimuth, CellTypist)’s tutorial.
2. Then, we mapped the annotation results with cell QC statistics and *ddqc* clustering ID using the cell barcodes.
3. Using the *table* function in R, we have calculated the intersections between ddqc initial cluster IDs and automatic annotations (Additional file 1: Table S5)
4. Finally, we did a run of *ddqc* on PBMC which used automatic annotations instead of clustering. It was done similar to the original *ddqc*, excluding the clustering step and grouping cells based on the independent annotation. Then the filtering cutoff was calculated for each group using MAD with the threshold of 2. Filtering was done on n_genes and percent_mito. We have compiled the results in Additional file 1: Table S5:
  - ddqc_cluster: the initial clustering ID from original ddqc
  - single_r, azimuth, cell_typist: automatic annotation
  - %method_name%_passed_qc: whether the cell passed the QC based on a particular grouping method

All methods (including the independent annotation and the original graph-based clustering using in *ddqc*) produced identical results.

We did a similar comparison for the Krasnow Lung dataset [47]. We only used CellTypist as SingleR did not have a training dataset for Human Lung, and Azimuth’s web interface had problems with processing this dataset. We evaluated results as for the PBMC dataset described above.

### Comparison of ddqc’s initial filtering for empty droplets and EmptyDrops

In order to assess robustness of the default Inital filtering in *ddqc* (cells with < 100 genes and > 80% mito are removed), we have compared it with EmptyDrops. We have performed our comparison using the steps below:

1. We downloaded the BAM and BAI files for Tabula Muris heart and lung dataset from their S3 storage bucket (https://s3.console.aws.amazon.com/s3/buckets/czb-tabula-muris-senis?prefix=10x/3_month/&region=us-west-2)
2. We ran EmptyDrops for Tabula Muris heart and lung datasets using the DropletUtils [77] R package and followed the directions in the vignette.
3. We have filtered out cells that had FDR more than 0.01 (as recommended in theEmptyDrops vignette). We have compared the results of this filtering with *ddqc*’s default by finding the number of common cells and cells that were retained by one method and not the other.

Afterwards, we ran *ddqc* on the EmptyDrops filtering. We then compared its results to regular *ddqc* with default filters by finding matches for each cluster among the other method clusters similar to the approach described in “Comparison of *ddqc* with independent cell classifiers section.”

### Variable MAD muliplier analysis

We analyzed the performance of *ddqc* by running it with different thresholds by varying the MAD multiplier. We ran *ddqc* for each threshold from 1 to 3.5 with 0.1 increments and recorded the number and percentage of cells filtered out for each cluster. We have also recorded other information, such as the cluster’s median, MAD, standard deviation, and MAD to SD ratio for n_counts, n_genes, and percent_mito.

Based on these results we have produced several plots:

- ggridges joyplot with rug broken down by cluster for each QC metric. Lines representing 1 * MAD (red), 2 * MAD (green), and 3 * MAD (blue) for each cluster were added to these plots in red, green, and blue colors respectively.
- Linechart with number or percentage of cells filtered on *y*-axis and threshold on *x*-axis faceted by cluster. (this plot was also included in the released version of Pegasus implementation of ddqc)

As part of this analysis, we have also predicted the modality of the distribution of QC metrics for each cluster. For Tabula Muris heart and lung, we have run the following functions:

- *dip.test* from diptest (https://cran.r-project.org/web/packages/diptest/index.html)
- *is.unimodal* from LaplacesDemon (https://cran.r-project.org/web/packages/LaplacesDemon/index.html)

For each cluster, we looked at n_counts, n_genes, and percent_mito and assessed whether it was unimodal. We have considered the cluster unimodal if *p*_value was less than 0.05 for diptest and if *is.unimodal* returned true for LaplacesDemon.

### Visualization and plotting

Boxplots, joyplots, and violin plots for each QC metric were generated in R using the *ggplot2* and *ggridges* packages. For the tissue summary plots (Fig. 1), only initial or empty droplet filtering was performed, and then the QC metrics plotted stratified by tissue. For cell-type summary plots (Fig. 2), graph-based clustering was performed after initial or empty droplet filtering. A horizontal red line for boxplots and violin plots, and vertical line for joyplots were added to illustrate standard cutoff thresholds (10% for % mitochondrial transcripts, 200 for number of genes).

All analysis tasks were performed on the Broad Institute High-Performance Computing Cluster.

## Supplementary Information

Additional file 1: Tables S1-S9. Supplementary tables.

Additional file 2. Supplementary Text [15, 35, 38, 47, 70–72, 84–91].

Additional file 3. Supplementary figure legends and supplementary figures.

Additional file 4. Review history.

## Data Availability

All datasets used in this manuscript are publicly available. Additional file 1: Tables S1 and S2 provide the information for accessing the datasets. *ddqc* is available as a Python package on GitHub along with a tutorial: https://github.com/ayshwaryas/ddqc [78], and on Zenodo (10.5281/zenodo.7297280 [79]). For R users, a compatible package is available on GitHub https://github.com/ayshwaryas/ddqc_R [80] and on Zenodo (10.5281/zenodo.7297276 [81]). The source code and all code used to generate the figures in the paper have been deposited on GitHub https://github.com/ayshwaryas/ddqc_source [82] and on Zenodo (10.5281/zenodo.7213410 [83]). All code is available for use under the open-source license BSD3.
