# Supplementary material for: Biology-inspired data-driven quality control for scientific discovery in single-cell transcriptomics
Source: Genome Biol. 2022 Dec 27;23:267. doi: 10.1186/s13059-022-02820-w (PMC9793662; doi:10.1186/s13059-022-02820-w)
Supplement: Supplementary file 2 — Additional file 2. Supplementary Text [15, 35, 38, 47, 70–72, 84–91]. [file 13059_2022_2820_MOESM2_ESM.docx]

**Additional File 2: SUPPLEMENTARY TEXT**

**Table 1 Description:** Quality control (QC) is typically performed for the following metrics: the number of UMIs (nUMI) detected, the number of genes (nGenes) detected, the fraction of mitochondrial genes (%mito), (sometimes the fraction of ribosomal genes (% ribo)), and the minimum number of cells in which a gene is present. Additionally, empty droplets or multiplets may be detected and removed, and ambient RNA accounted for. We categorized the 107 studies into groups:

1. Papers with any QC: Where any QC was performed on the QC metrics
2. Data-agnostic fixed threshold: The majority of the studies use a single threshold - for example <10% mitochondrial transcripts.
3. Multiple fixed thresholds varying by sample: Sometimes, different fixed cutoffs are used for different samples within one study.
4. Mitochondrial or ribosomal genes removed before analysis: Sometimes studies regress out or remove mitochondrial and ribosomal genes.
5. Data-driven study-level thresholds: Some studies used “data-driven” cutoffs (Data-Driven study-level threshold) - for example within 2 SDs from the median performed on a per-sample basis or custom cut-offs that are very specific to the scientific question in which they are used.
6. Custom QC: Some studies came up with custom QC for the unique features of their data or samples.

**Survey of QC practices suggests a need for data-driven QC (misc)**

While most studies used data-agnostic QC filters (**Table 1, Additional File 1:Table S1**), QC filter thresholds also varied with protocols (cells or nuclei) or technology (10x v3 vs v2 chemistry)[84] Some studies excluded ribosomal protein or mitochondrial genes altogether or had a cutoff on the fraction of ribosomal genes[85]. Custom QC metrics were also adopted such as transcriptome mappability rates to exon vs non gene bodies [86] or the fraction of reads mapping to housekeeping [87] or select other genes such as *KCNQ1OT1*[88], actin[38] or Hemoglobin [89] Some studies had incorporated custom data-driven approaches including probabilistic mixture models [15,90] or sample [90,91] and study specific filters[35], suggesting the awareness and need for generalizable data-driven QC approaches.

**Comparison of clustering algorithms on *ddqc* results**

We compared the performance of *ddqc* across six clustering algorithms on two case study datasets: (1) the Tabula Muris Heart and Aorta tissue and (2) the Tabula Muris lung tissue dataset as a benchmark: (1) louvain, (2) leiden, (3) spectral_louvain, (4) spectral_leiden, (5) k-means clustering, (6) hierarchical clustering, where algorithms 1-4 are provided by Pegasus library, algorithms 5 and 6 implementations is described in the Methods section. For all algorithms, default parameters were used.

In this head-to-head comparison, we show that all algorithms produced nearly identical results (**Additional File 1: Table S4, Additional File 3:Fig. S4 A,B**) on the two case study datasets. We have compared the barcodes of cells retained by different algorithms. In the heart and aorta dataset, 2568 recovered cell barcodes post *ddqc* are shared among all clustering algorithms, with the final number of cells ranging from 2571(hierarchical) to 2587 (Leiden). Similar results are observed in lung, where 15601 barcodes are shared, with the final number of barcodes ranging from 15682 (k-means) to 15892 (Leiden). In the heart, Louvain (retaining 2586 cells) performed on par with Leiden and better than other clustering methods. In lung, Leiden was the only cluster which retained more cells (40 additional pericyte cells) which were removed in the default Louvain clustering method (based on the QC metric of number of UMI counts).

The clustering algorithm is a user-adjustable argument in the *ddqc* Python package for the four options which performed in general better than k-means or hierarchical (1) louvain, (2) leiden, (3) spectral_louvain, (4) spectral_leiden, and updated the tutorial to reflect these changes.

**Comparison of pipeline hyperparameters on *ddqc* results**

We performed a benchmark of varying hyperparameters for three steps (Principal Component Analysis, number of neighbors for K-NN graph inference, Clustering resolution) for 12 combinations on the Tabula Muris (1) Heart and Aorta and (2) lung datasets as a case study. The combinations are as below:

Number of PCs, Number of neighbors (K), Clustering resolution

1. Default: 50, 20, 1.4

changing only resolution:

1. 50,20,1
2. 50,20,0.5

changing K and resolution:

1. 50,10,1.4
2. 50,10,1
3. 50,10,0.5

changing number of PCs and resolution:

1. 20,20,1.4
2. 20,20,1
3. 20,20,0.5

changing number of PCs, K, and resolution:

1. 20,10,1.4
2. 20,10,1
3. 20,10,0.5

We find that the results from our default options remain robust. The results are summarized in supplementary figure panels S4C and S4D.

**Comparison of *ddqc* on clustering vs automated annotation to define cell types**

We independently annotated cells of the PBMC dataset using three different automated annotation methods including both classification and non-classification-based strategies:

SingleR (<https://bioconductor.org/packages/release/bioc/html/SingleR.html>) [71]

Azimuth (<https://azimuth.hubmapconsortium.org>) [72]

CellTypist (<https://www.celltypist.org/>) [70]

Each of these methods assigns a cell type to each cell (we note that neither of these independent approaches throws out any cells as “unannotable” regardless of quality suggesting some inherent

bias/inaccuracy in the approaches themselves). Thus, we had three independent annotations for

each cell (by each of these methods). In contrast, *ddqc* uses clustering to dissect biological heterogeneity and group cells into clusters, representing their approximate biological cell type. We mapped *ddqc* clusters with independent annotations and note that *ddqc* cluster IDs had high correspondence to independent annotation groups.

*ddqc* applies MAD-based filtering on clusters. To compare the results of independent classification with that from *ddqc*, we grouped cells with the same automated cell-type annotation and applied MAD-based filtering on each such group, independently for each of the methods.

**Additional File 1: Table S5** provides a mapping between *ddqc* results post clustering and post each of the independent classification methods. A comparison of cells removed by MAD-based filtering revealed no differences between independent classification and clustering-based approaches. In all cases, the same 6 cells were filtered out highlighting that clustering performs on par with other independent methods for the purposes of *ddqc*.

To compare a second independent example, we compared the human lung dataset[47] which has served as a use case in **Additional File 3: Fig. S5** of the manuscript. Azimuth unfortunately had processing issues; and hence, we only use CellTypist for this comparison. Table S5 provides a mapping between *ddqc* results after clustering and each of the independent classification methods.

We note that both methods retain 115,591 cells comprising 98.9% of *ddqc* results and 99.9% of CellTypist results, showing high concordance. Summary of results:

- Total number of cells in Krasnow Lung after initial QC (> 100 genes, < 80% mito): 122,301
- Cells that are retained by *ddqc* post clustering: 116,854
- Cells that are retained by *ddqc* post CellTypist: 115,672
- Intersection of *ddq*c post clustering and post CellTypist: 115,591 (98.9% of *ddqc* results; 99.9% of CellTypist results)
- Cells that are uniquely retained by *ddqc* post clustering: 1263 (1% of input)
- Cells that are unique retained by *ddqc* post CellTypist: 81 (0.06% of input)

We attempted to apply independent classification methods to datasets besides the PBMC and lung examples, however, all three approaches (SingleR, Azimuth, CellTypist) are currently limited in the range of tissues they can annotate and only provide capabilities for ~5 example organs, mostly human data. Otherwise, one must provide a well-annotated training or reference dataset. Based on our comparisons of PBMC and human lung datasets, and in consideration of the above

limitations, we conclude that clustering is a reasonable method to use until reference atlases for multiple tissues are curated by the community and independent classification methods become routine and well tested which we anticipate will take a few years.

**Assessment of *ddqc* adaptive threshold MAD multiplier**

To evaluate if different clusters within a dataset vary in the spread and distributions of their QC metrics, we first confirmed that the density distribution of the QC metrics were largely unimodal by running the following functions:

- *dip.test()* from the R package diptest (<https://cran.r-project.org/web/packages/diptest/index.html>)
- *is.unimodal()* from the R package LaplacesDemon (<https://cran.r-project.org/web/packages/LaplacesDemon/index.html>)

For each cluster we looked at n_counts, n_genes, and percent_mito and assessed whether it was unimodal. We considered the cluster as unimodal if p_value was less than 0.05 for *diptest* and if *is.unimodal* returned *TRUE* for *LaplacesDemon*. We observed that the majority of the clusters in our test datasets were unimodal (nUMI: 79.4-94%, nGene: 73-89.25%, percent.mito: 76-83%) validating an approach that filters off the extreme tail (over a mixture model assuming explicit number of modes).

We next observed the trend of the number of cells filtered as we sweep along the MAD multiplier. We note that the trend varies from cluster to cluster but that overall, a MAD multiplier of 2 is robust across trends and chosen as the default value. We also provide the user with interactive plots to provide the MAD multiplier as an argument.
