# Supplementary material for: Biology-inspired data-driven quality control for scientific discovery in single-cell transcriptomics
Source: Genome Biol. 2022 Dec 27;23:267. doi: 10.1186/s13059-022-02820-w (PMC9793662; doi:10.1186/s13059-022-02820-w)
Supplement: Supplementary file 3 — Additional file 3. Supplementary figure legends and supplementary figures. [file 13059_2022_2820_MOESM3_ESM.pdf]

Additional File 3: Supplementary Figures and legends

Fig. S1: QC metrics vary by tissue: Fraction of mitochondrial reads (A,B), gene complexity (C,D) and percentage of ribosomal protein genes (E,F) per cell across mouse tissues and technologies. Various mouse tissue scRNAseq datasets generated in the *Tabula muris* project by 10X droplet-based (A,C,E) and Smart-seq2 (SS2; B,D,F) plate-based technologies. Each row in a panel is a density curve with the mean represented by a blue diamond. Red lines indicate conventional threshold values set at 10% for percentage of mitochondrial reads, and 200 for gene complexity.

Fig. S2: QC metrics vary by tissue: Fraction of mitochondrial reads (A,B), gene complexity (C,D) and percentage of ribosomal protein genes (E,F) per cell across mouse tissues and cancers. Various mouse tissue scRNAseq datasets generated in the *Tabula senis* project (30 months) (A,C,E) and the human tumor atlas pilot project (HTAPP; B,D,F) by 10X droplet-based. Each row in a panel is a density curve with the mean represented by a blue diamond. Red lines indicate conventional threshold values set at 10% for percentage of mitochondrial reads, and 200 for gene complexity.

Fig. S3: QC metrics vary by cell-type: Fraction of mitochondrial reads (A,B), gene complexity (C,D) and percentage of ribosomal protein genes (E,F) per cell across cell types of various mouse tissues: Cerebellum (A), colon (B), mammary gland (C), lung (D), tongue (E) and Lung (F). All scRNA-seq data was generated using the 10X droplet-based technology. Each row in a panel is a density curve with the mean represented by a blue diamond. Red lines indicate conventional threshold values set at 10% for percentage of mitochondrial reads, and 200 for gene complexity. Cluster numbers are indicated preceding the cell type annotation.

Fig. S4: (A, B) Impact of varying the clustering algorithm (hierarchical, k-means, leiden, spectral-leiden, louvain, spectral-louvain) on *ddqc* results. UpsetR visualization of barcodes retained by *ddqc* for each clustering algorithm applied to the *Tabula Muris* Heart and Aorta (A) and lung (B) datasets. Vertical bar height represents the number of retained barcodes shared among clustering methods indicated by line segment. Horizontal bars represent the total number of barcodes retained by *ddqc* after each clustering algorithm. (C,D) Impact of varying the hyperparameters (louvain clustering resolution - number of principal components - number of neighbors K used in KNN graph inference) on *ddqc* results. UpsetR visualization of barcodes retained by *ddqc* for combination of hyperparameters applied to the *Tabula Muris* Heart and Aorta (C) and lung (D) datasets. Vertical bar height represents the number of retained barcodes shared among combinations indicated by line segment. Horizontal bars represent the total number of barcodes retained by *ddqc* after each combination. (E-H) *ddqc* retains cell states of biological relevance. Boxplot visualization of the fraction of reads mapping to the mitochondria, and the gene complexity across cell types in the (E, G) mouse heart and aorta and (F,H) human olfactory epithelium.

Fig. S5: (A-D) *ddqc* retains cell states of biological relevance. UMAP visualization of joint clustering of cells retained by both *ddqc* and the standard cutoff in (A) human olfactory epithelium and (C) human lung. Proportion of cells retained by *ddqc*, standard cutoff or both in (B) human olfactory epithelium and (D) human lung. (E, F) Comparison of *ddqc* and miQC results. UMAP visualization of cells retained by both *ddqc* and miQC on (E) *Tabula Muris* Heart and Aorta and (F) Human Olfactory Epithelium.

Figure S1

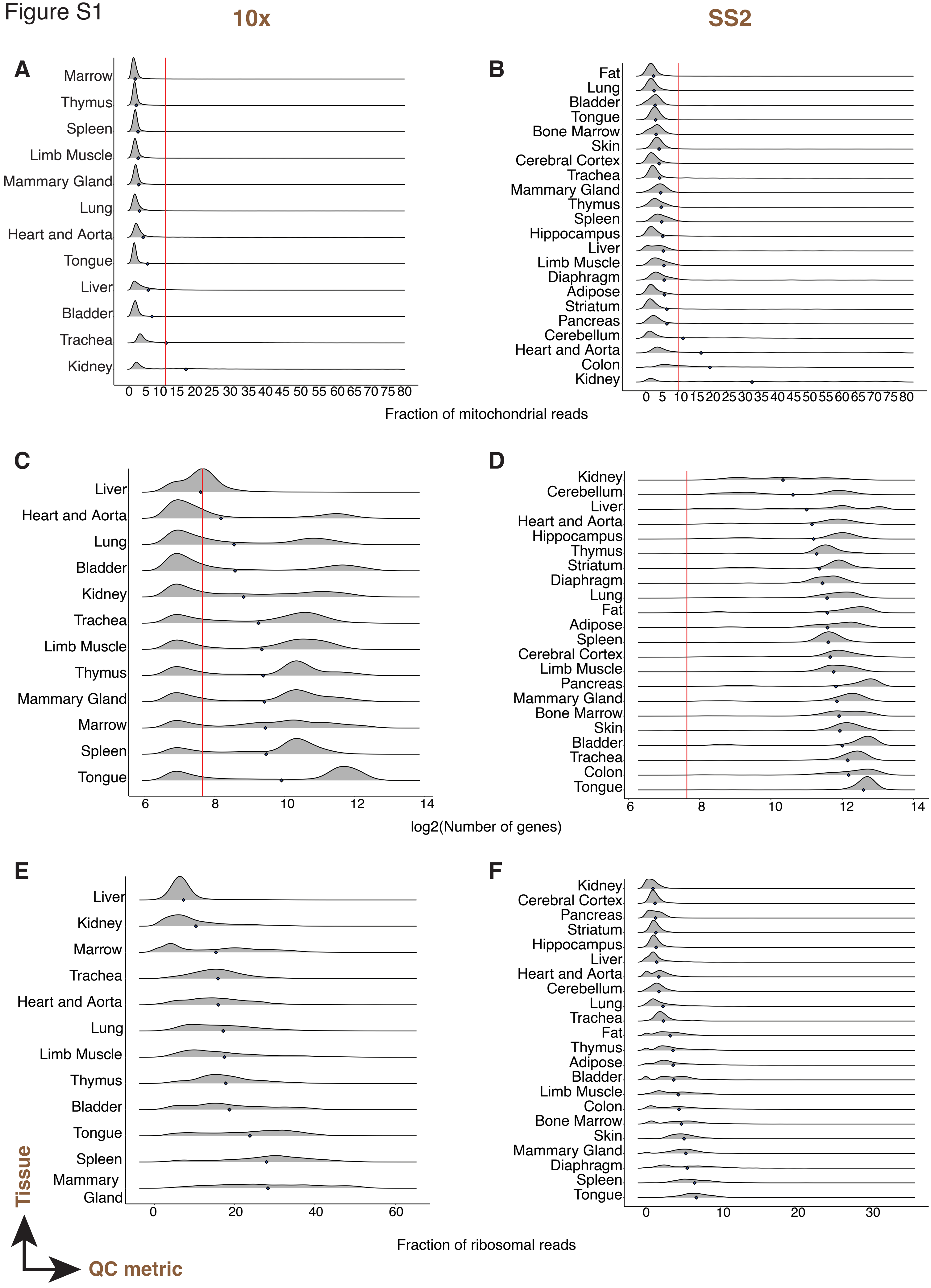
10x
SS2
A
Marrow
Thymus
Spleen
Limb Muscle
Mammary Gland
Lung
Heart and Aorta
Tongue
Liver
Bladder
Trachea
Kidney
0 5 10 15 20 25 30 35 40 45 50 55 60 65 70 75 80
B
Fat
Lung
Bladder
Tongue
Bone Marrow
Skin
Cerebral Cortex
Trachea
Mammary Gland
Thymus
Spleen
Hippocampus
Liver
Limb Muscle
Diaphragm
Adipose
Striatum
Pancreas
Cerebellum
Heart and Aorta
Colon
Kidney
0 5 10 15 20 25 30 35 40 45 50 55 60 65 70 75 80
Fraction of mitochondrial reads
C
Liver
Heart and Aorta
Lung
Bladder
Kidney
Trachea
Limb Muscle
Thymus
Mammary Gland
Marrow
Spleen
Tongue
6 8 10 12 14
D
Kidney
Cerebellum
Liver
Heart and Aorta
Hippocampus
Thymus
Striatum
Diaphragm
Lung
Fat
Adipose
Spleen
Cerebral Cortex
Limb Muscle
Pancreas
Mammary Gland
Bone Marrow
Skin
Bladder
Trachea
Colon
Tongue
6 8 10 12 14
log2(Number of genes)
E
Liver
Kidney
Marrow
Trachea
Heart and Aorta
Lung
Limb Muscle
Thymus
Bladder
Tongue
Spleen
Mammary Gland
0 20 40 60
F
Kidney
Cerebral Cortex
Pancreas
Striatum
Hippocampus
Liver
Heart and Aorta
Cerebellum
Lung
Trachea
Fat
Thymus
Adipose
Bladder
Limb Muscle
Colon
Bone Marrow
Skin
Mammary Gland
Diaphragm
Spleen
Tongue
0 10 20 30
Fraction of ribosomal reads
Tissue
QC metric

Figure S2

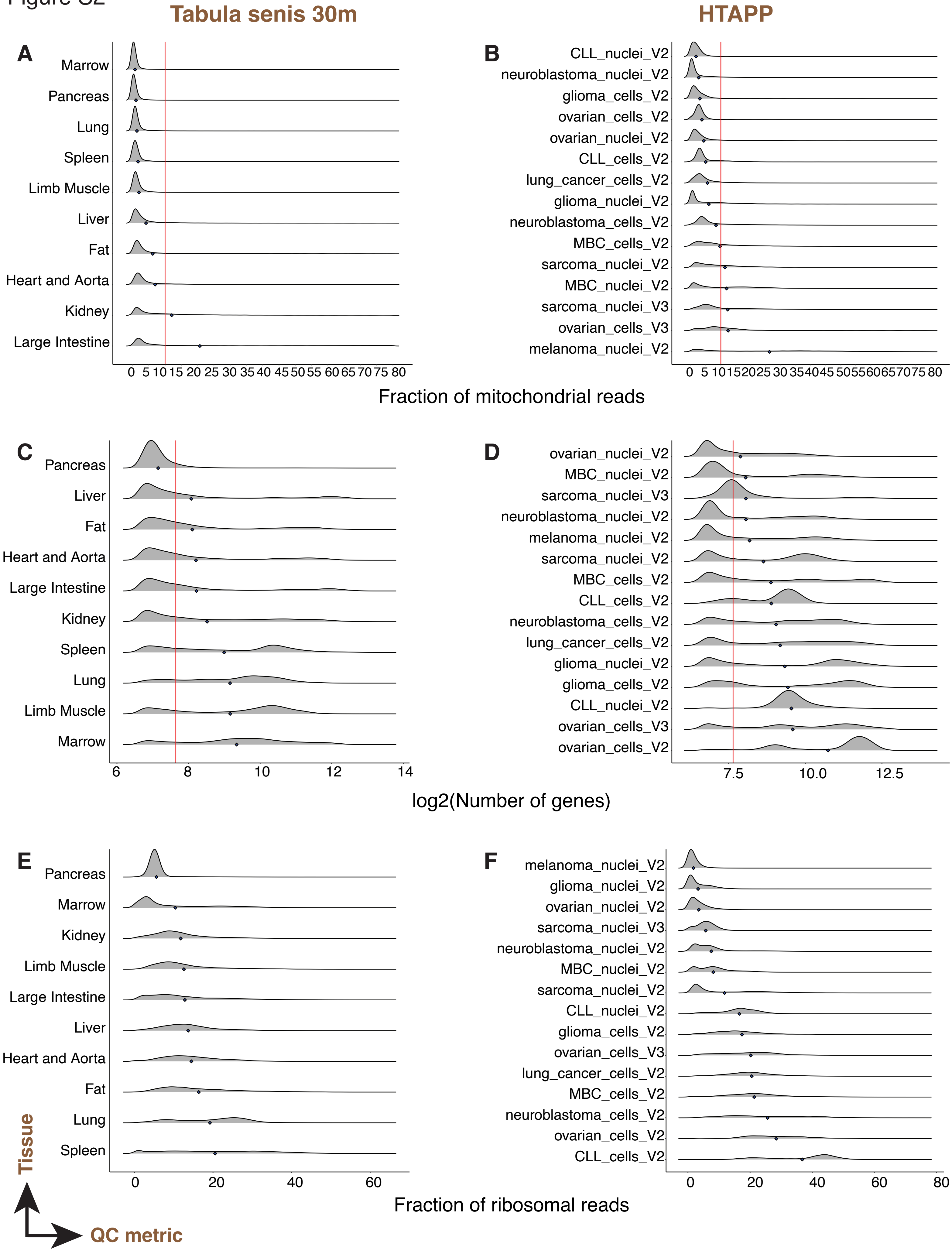

Tabula senis 30m
HTAPP
A
Marrow
Pancreas
Lung
Spleen
Limb Muscle
Liver
Fat
Heart and Aorta
Kidney
Large Intestine
0 5 10 15 20 25 30 35 40 45 50 55 60 65 70 75 80
B
CLL_nuclei_V2
neuroblastoma_nuclei_V2
glioma_cells_V2
ovarian_cells_V2
ovarian_nuclei_V2
CLL_cells_V2
lung_cancer_cells_V2
glioma_nuclei_V2
neuroblastoma_cells_V2
MBC_cells_V2
sarcoma_nuclei_V2
MBC_nuclei_V2
sarcoma_nuclei_V3
ovarian_cells_V3
melanoma_nuclei_V2
0 5 10152025 30 354045 5055606570 75 80
Fraction of mitochondrial reads
C
Pancreas
Liver
Fat
Heart and Aorta
Large Intestine
Kidney
Spleen
Lung
Limb Muscle
Marrow
6 8 10 12 14
D
ovarian_nuclei_V2
MBC_nuclei_V2
sarcoma_nuclei_V3
neuroblastoma_nuclei_V2
melanoma_nuclei_V2
sarcoma_nuclei_V2
MBC_cells_V2
CLL_cells_V2
neuroblastoma_cells_V2
lung_cancer_cells_V2
glioma_nuclei_V2
glioma_cells_V2
CLL_nuclei_V2
ovarian_cells_V3
ovarian_cells_V2
7.5 10.0 12.5
log2(Number of genes)
E
Pancreas
Marrow
Kidney
Limb Muscle
Large Intestine
Liver
Heart and Aorta
Fat
Lung
Spleen
0 20 40 60
F
melanoma_nuclei_V2
glioma_nuclei_V2
ovarian_nuclei_V2
sarcoma_nuclei_V3
neuroblastoma_nuclei_V2
MBC_nuclei_V2
sarcoma_nuclei_V2
CLL_nuclei_V2
glioma_cells_V2
ovarian_cells_V3
lung_cancer_cells_V2
MBC_cells_V2
neuroblastoma_cells_V2
ovarian_cells_V2
CLL_cells_V2
0 20 40 60 80
Fraction of ribosomal reads
Tissue
QC metric

Figure S3

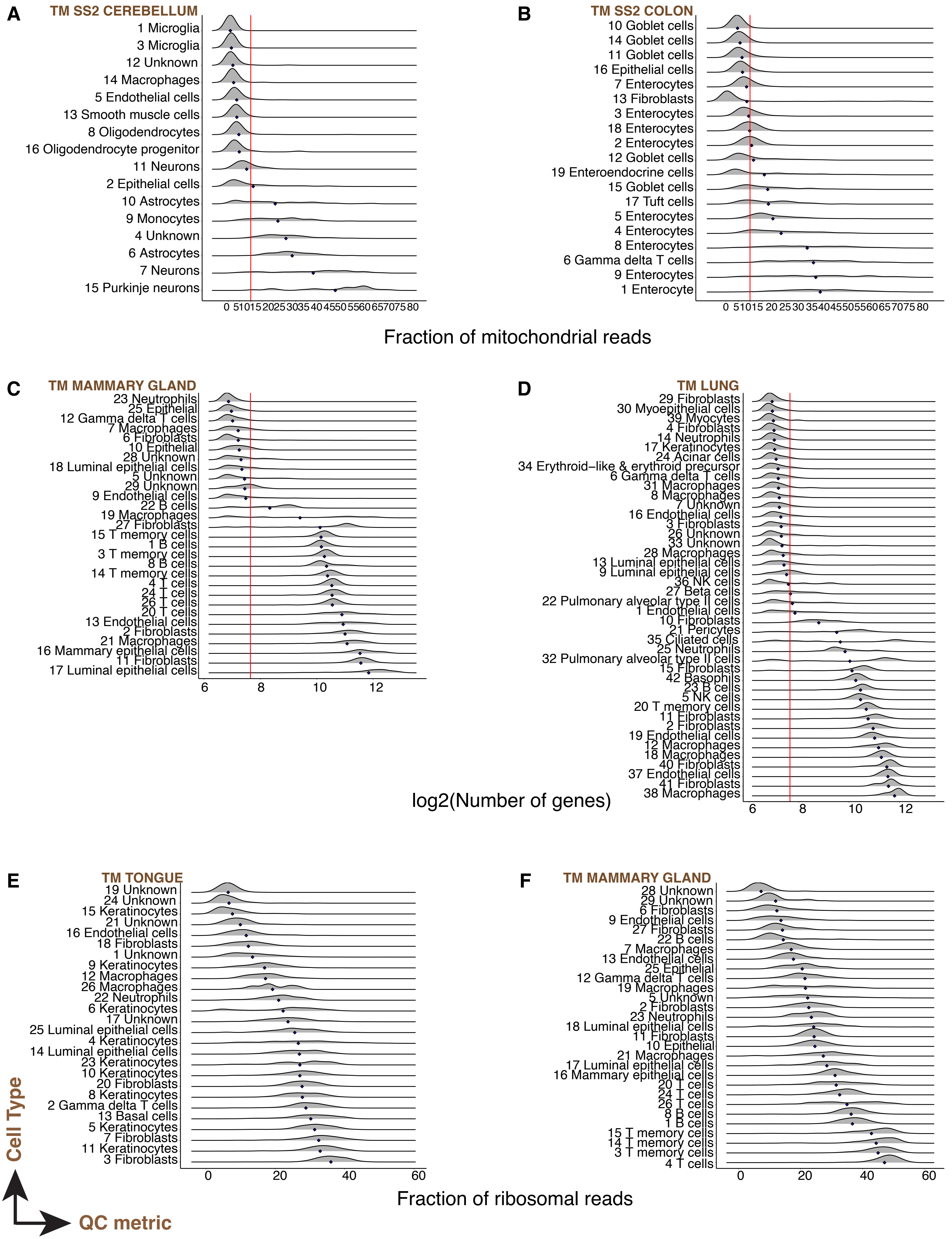
A
TM SS2 CEREBELLUM
1 Microglia
3 Microglia
12 Unknown
14 Macrophages
5 Endothelial cells
13 Smooth muscle cells
8 Oligodendrocytes
16 Oligodendrocyte progenitor
11 Neurons
2 Epithelial cells
10 Astrocytes
9 Monocytes
4 Unknown
6 Astrocytes
7 Neurons
15 Purkinje neurons
0 5 10 15 20 25 30 35 40 45 50 55 60 65 70 75 80
B
TM SS2 COLON
10 Goblet cells
14 Goblet cells
11 Goblet cells
16 Epithelial cells
7 Enterocytes
13 Fibroblasts
3 Enterocytes
18 Enterocytes
2 Enterocytes
12 Goblet cells
19 Enteroendocrine cells
15 Goblet cells
17 Tuft cells
5 Enterocytes
4 Enterocytes
8 Enterocytes
6 Gamma delta T cells
9 Enterocytes
1 Enterocyte
0 5 10 15 20 25 30 35 40 45 50 55 60 65 70 75 80
Fraction of mitochondrial reads
C
TM MAMMARY GLAND
23 Neutrophils
25 Epithelial
12 Gamma delta T cells
7 Macrophages
6 Fibroblasts
10 Epithelial
28 Unknown
18 Luminal epithelial cells
5 Unknown
29 Unknown
9 Endothelial cells
22 B cells
19 Macrophages
27 Fibroblasts
15 T memory cells
1 B cells
3 T memory cells
8 B cells
14 T memory cells
4 T cells
24 T cells
26 T cells
20 T cells
13 Endothelial cells
2 Fibroblasts
21 Macrophages
16 Mammary epithelial cells
11 Fibroblasts
17 Luminal epithelial cells
6 8 10 12
D
TM LUNG
29 Fibroblasts
30 Myoepithelial cells
39 Myocytes
4 Fibroblasts
14 Neutrophils
17 Keratinocytes
24 Acinar cells
34 Erythroid−like & erythroid precursor
6 Gamma delta T cells
31 Macrophages
8 Macrophages
7 Unknown
16 Endothelial cells
3 Fibroblasts
26 Unknown
33 Unknown
28 Macrophages
13 Luminal epithelial cells
9 Luminal epithelial cells
36 NK cells
27 Beta cells
22 Pulmonary alveolar type II cells
1 Endothelial cells
10 Fibroblasts
21 Pericytes
35 Ciliated cells
25 Neutrophils
32 Pulmonary alveolar type II cells
15 Fibroblasts
42 Basophils
23 B cells
5 NK cells
20 T memory cells
11 Fibroblasts
2 Fibroblasts
19 Endothelial cells
12 Macrophages
18 Macrophages
40 Fibroblasts
37 Endothelial cells
41 Fibroblasts
38 Macrophages
6 8 10 12
log2(Number of genes)
E
TM TONGUE
19 Unknown
24 Unknown
15 Keratinocytes
21 Unknown
16 Endothelial cells
18 Fibroblasts
1 Unknown
9 Keratinocytes
12 Macrophages
26 Macrophages
22 Neutrophils
6 Keratinocytes
17 Unknown
25 Luminal epithelial cells
4 Keratinocytes
14 Luminal epithelial cells
23 Keratinocytes
10 Keratinocytes
20 Fibroblasts
8 Keratinocytes
2 Gamma delta T cells
13 Basal cells
5 Keratinocytes
7 Fibroblasts
11 Keratinocytes
3 Fibroblasts
0 20 40 60
F
TM MAMMARY GLAND
28 Unknown
29 Unknown
6 Fibroblasts
9 Endothelial cells
27 Fibroblasts
22 B cells
7 Macrophages
13 Endothelial cells
25 Epithelial
12 Gamma delta T cells
19 Macrophages
5 Unknown
2 Fibroblasts
23 Neutrophils
18 Luminal epithelial cells
11 Fibroblasts
10 Epithelial
21 Macrophages
17 Luminal epithelial cells
16 Mammary epithelial cells
20 T cells
24 T cells
26 T cells
8 B cells
1 B cells
15 T memory cells
14 T memory cells
3 T memory cells
4 T cells
0 20 40 60
Fraction of ribosomal reads
Cell Type
QC metric

Figure S4

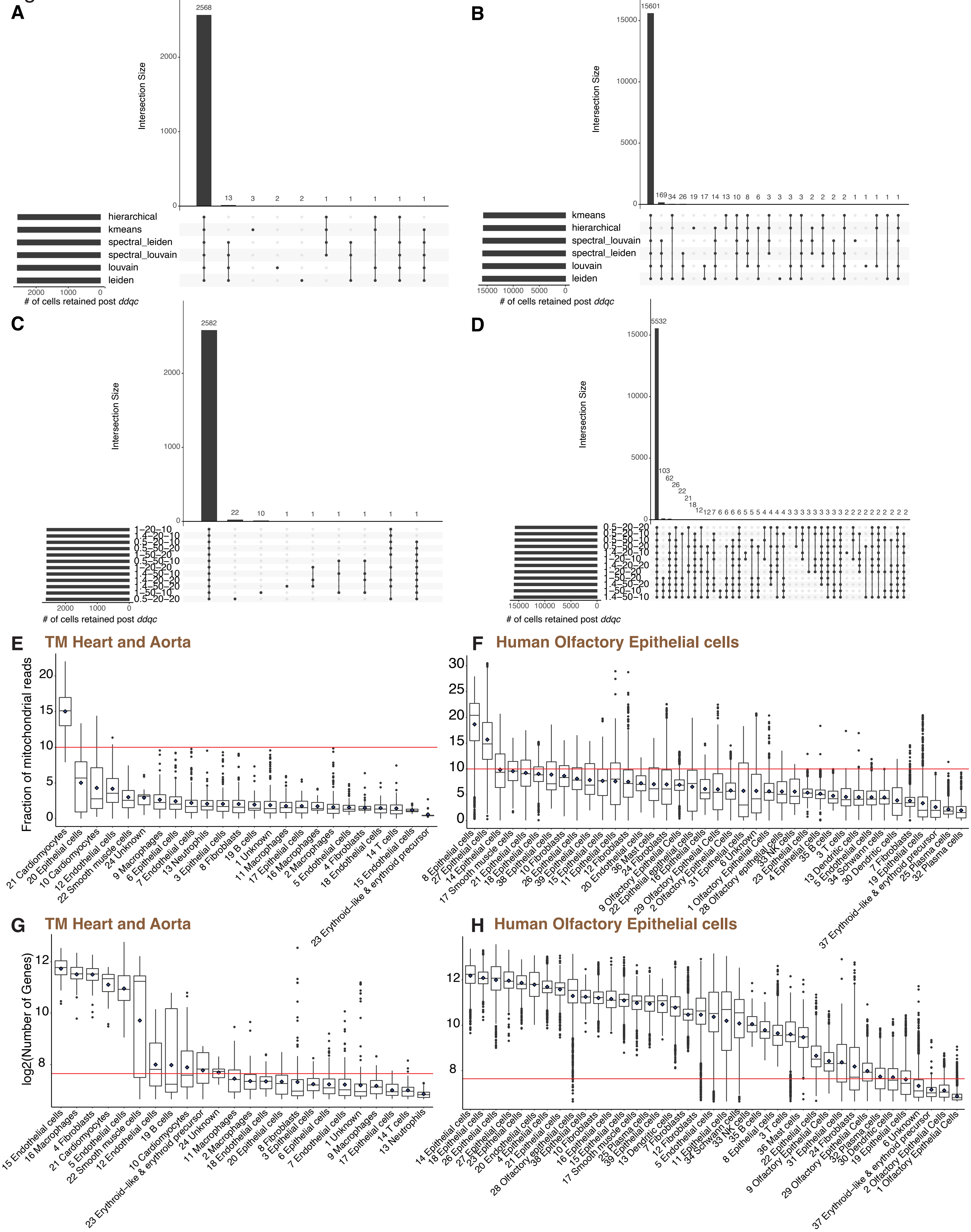

A
Intersection Size
2568
13
3
2
2
1
1
1
1
1
hierarchical
kmeans
spectral_leiden
spectral_louvain
louvain
leiden
# of cells retained post ddqc
B
Intersection Size
15601
169
kmeans
hierarchical
spectral_louvain
spectral_leiden
louvain
leiden
# of cells retained post ddqc
C
Intersection Size
2582
22
10
# of cells retained post ddqc
D
Intersection Size
5532
103
62
26
22
21
18
# of cells retained post ddqc
E
TM Heart and Aorta
Fraction of mitochondrial reads
F
Human Olfactory Epithelial cells
G
TM Heart and Aorta
log2(Number of Genes)
H
Human Olfactory Epithelial cells

Figure S5

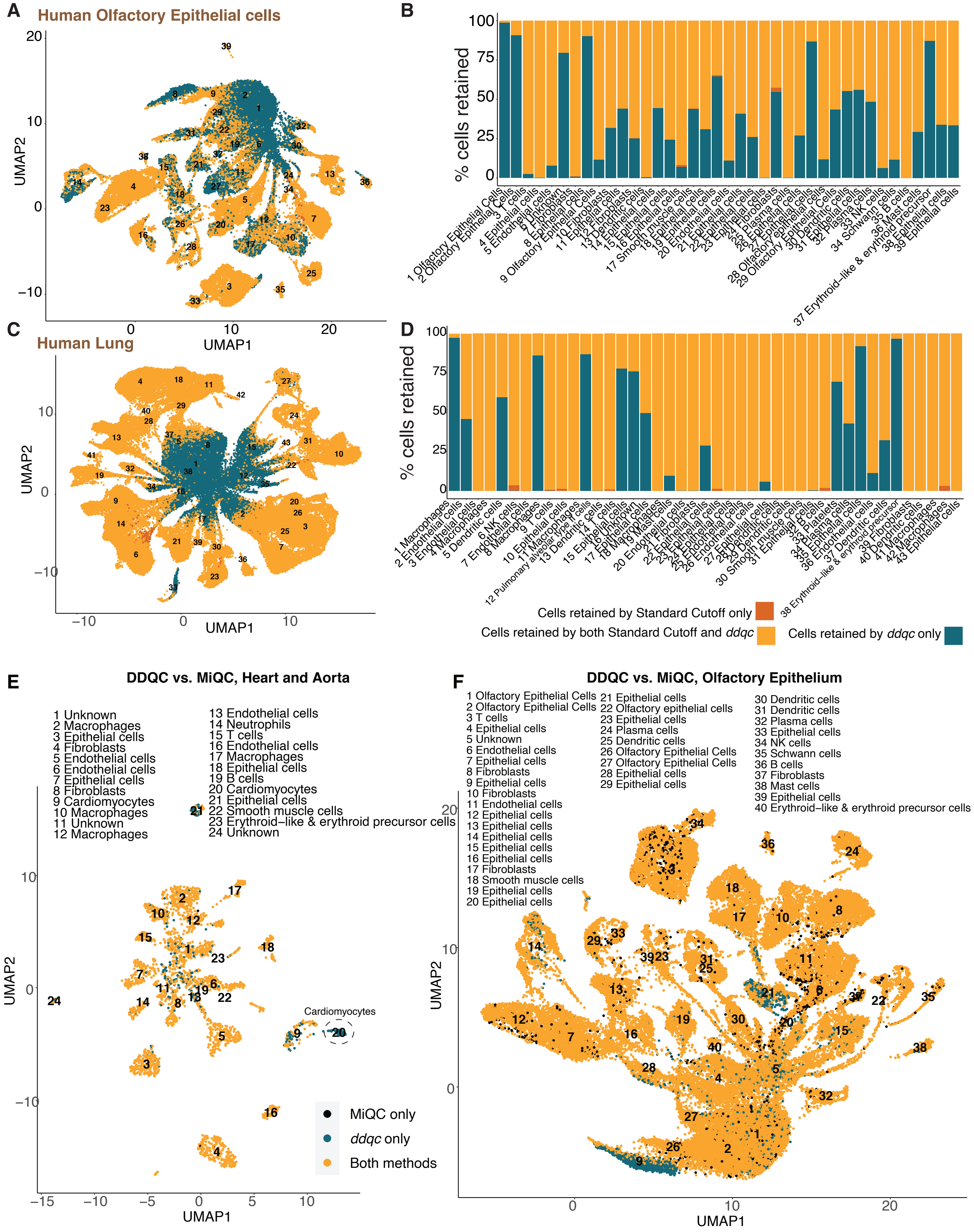
A
Human Olfactory Epithelial cells
UMAP1
UMAP2
B
% cells retained
1 Olfactory Epithelial Cells
2 Olfactory Epithelial Cells
3 T cells
4 Epithelial cells
5 Endothelial cells
6 Unknown
7 Fibroblasts
8 Epithelial cells
9 Olfactory Epithelial Cells
10 Fibroblasts
11 Epithelial cells
12 Fibroblasts
13 Dendritic cells
14 Epithelial cells
15 Epithelial cells
16 Epithelial cells
17 Smooth muscle cells
18 Epithelial cells
19 Epithelial cells
20 Endothelial cells
21 Epithelial cells
22 Epithelial cells
23 Epithelial cells
24 Fibroblasts
25 Plasma cells
26 Epithelial cells
27 Olfactory epithelial cells
28 Olfactory Epithelial Cells
29 Olfactory Epithelial Cells
30 Dendritic cells
31 Epithelial cells
32 Plasma cells
33 NK cells
34 Schwann cells
35 B cells
36 Mast cells
37 Erythroid–like & erythroid precursor
38 Epithelial cells
39 Epithelial cells
C
Human Lung
UMAP1
UMAP2
D
% cells retained
1 Macrophages
2 Endothelial cells
3 Endothelial cells
4 Macrophages
5 Dendritic cells
6 NK cells
7 Endothelial cells
8 Macrophages
9 T cells
10 Epithelial cells
11 Macrophages
12 Pulmonary alveolar type II cells
13 Dendritic cells
14 T cells
15 Epithelial cells
16 Unknown
17 Epithelial cells
18 Macrophages
19 Mast cells
20 Endothelial cells
21 Fibroblasts
22 Epithelial cells
23 Epithelial cells
24 Epithelial cells
25 Endothelial cells
26 Endothelial cells
27 Epithelial cells
28 Dendritic cells
29 Dendritic cells
30 Smooth muscle cells
31 Epithelial cells
32 B cells
33 Platelets
34 Plasma cells
35 Epithelial cells
36 Endothelial cells
37 Dendritic cells
38 Erythroid–like & erythroid precursor
39 Fibroblasts
40 Dendritic cells
41 Macrophages
42 Macrophages
43 Epithelial cells
Cells retained by Standard Cutoff only
Cells retained by both Standard Cutoff and ddqc
Cells retained by ddqc only
E
DDQC vs. MiQC, Heart and Aorta
1 Unknown
2 Macrophages
3 Epithelial cells
4 Fibroblasts
5 Endothelial cells
6 Endothelial cells
7 Epithelial cells
8 Fibroblasts
9 Cardiomyocytes
10 Macrophages
11 Unknown
12 Macrophages
13 Endothelial cells
14 Neutrophils
15 T cells
16 Endothelial cells
17 Macrophages
18 Epithelial cells
19 B cells
20 Cardiomyocytes
21 Epithelial cells
22 Smooth muscle cells
23 Erythroid–like & erythroid precursor cells
24 Unknown
Cardiomyocytes
MiQC only
ddqc only
Both methods
UMAP1
UMAP2
F
DDQC vs. MiQC, Olfactory Epithelium
1 Olfactory Epithelial Cells
2 Olfactory Epithelial Cells
3 T cells
4 Epithelial cells
5 Unknown
6 Endothelial cells
7 Epithelial cells
8 Fibroblasts
9 Epithelial cells
10 Fibroblasts
11 Endothelial cells
12 Epithelial cells
13 Epithelial cells
14 Epithelial cells
15 Epithelial cells
16 Epithelial cells
17 Fibroblasts
18 Smooth muscle cells
19 Epithelial cells
20 Epithelial cells
21 Epithelial cells
22 Olfactory epithelial cells
23 Epithelial cells
24 Plasma cells
25 Dendritic cells
26 Olfactory Epithelial Cells
27 Olfactory Epithelial Cells
28 Epithelial cells
29 Epithelial cells
30 Dendritic cells
31 Dendritic cells
32 Plasma cells
33 Epithelial cells
34 NK cells
35 Schwann cells
36 B cells
37 Fibroblasts
38 Mast cells
39 Epithelial cells
40 Erythroid–like & erythroid precursor cells
UMAP1
UMAP2
