# Supplementary material for: Biology-inspired data-driven quality control for scientific discovery in single-cell transcriptomics
Source: Genome Biol. 2022 Dec 27;23:267. doi: 10.1186/s13059-022-02820-w (PMC9793662; doi:10.1186/s13059-022-02820-w)
Supplement: Supplementary file 4 — Additional file 4. Review history. [file 13059_2022_2820_MOESM4_ESM.docx]

Review History

**First round of review**

**Reviewer 1**

**Were you able to assess all statistics in the manuscript, including the appropriateness of statistical tests used?**

Yes, and I have assessed the statistics in my report.

**Comments to author:**

The authors showed that QC metrics in scRNA-seq data vary with biological factors. Based on their findings, they propose ddqc, a data-driven QC filtering method. They found that ddqc can find cell-type clusters missed by the conventional data-agnostic method. The effectiveness of ddqc has been validated using many data sets, and the manuscript is expected to be beneficial to the single-cell biology community.

1. In the graph-based clustering step, if a cluster of low-quality cells is generated, such cells may be left behind. Is it possible to evaluate the accuracy of this point more quantitatively by simulated data?

2. There are a number of hyperparameters in ddqc (e.g., number of principal components, the K and the resolution in the graph-based clustering steps). Can these values be determined independently of the underlying biological variation? It would be great if the author discuss the effect of the choice of these hyperparameters on the results.

3. For Fig. 1 and 2, please specify in the figure legend how the QC metrics were aggregated (average, median, etc.) across cells in "each" study to derive the distribution "across" studies.

4. Please write in the legend of Fig. 2 what the numbers in the y axis label mean.

5. [p. 12 line 4] The results of the comparison with minQC should be presented not only as sentences but also as a Supplementary Figure or Supplementary Table.

6. Regarding Code Availability, it would be appreciated if the requirement for R version is stated in the README on the GitHub repository (I failed to install the R package possibly due to the old version R).

**Reviewer 2**

**Were you able to assess all statistics in the manuscript, including the appropriateness of statistical tests used?**

Yes, and I have assessed the statistics in my report.

**Comments to author:**

Subramanian et al. present an approach which employs a dynamic 'data driven' solution for determining QC cut-offs for single cell data. The paper describes a significant challenge in single cell analysis, and one that is often ignored or unrealised. It remains normal to apply 'commonly used' QC thresholds irrespective of the type of cells or taking into account other biologically relevant information. Indeed, the field seems to have almost accepted this, with deviations from 'common' thresholds challenged. The consequence is that many studies likely introduce some sort of bias, either though exclusion of cells / data that is meaningful or inclusion of background noise. Separating the two and dynamically drawing the correct line is challenging. The introduction is particularly good at outlining the issues, and providing some specific but well understood biological examples that underscore the necessity to develop better methods.

1.	The argument is made that data must be partitioned by biology and QC performed on those partitions. The concern that I have is that this partitioning rests completely on clustering (graph-based), with all cells 'clustering' together then arbitrarily considered as a the same and QC applied to each cluster separately. We already know that clustering is imperfect, with different cell types clustering together, and different patterns of 'noise' imitating real cell types and clustering together. My interpretation is that the problem ddqc is aiming to solve for a 'single sample' are the same for a subset of cells in a sample. Moving one level down the resolution helps somewhat, but still doesn't solve the underlying issue completely. To help demonstrate utility, it would be valuable to show performance on clusters, where cells are also independently classified, and thus your ability to show removal of poor QC cells and retention of real cells. PBMCs would be useful given the ability to accurately classify cells, and identify cells/droplets that do not align to a reference cell type. The later are hopefully filtered by ddqc and the former kept. Showing these results would be valuable.

2.	It would also be helpful to demonstrate robustness and similar results across multiple clustering methods. This could easily be incorporated into the package.

3.	At a simplistic level, ddqc appears to apply MAD on a cluster on a single parameter (QC metric). It would help to make a stronger case for the novelty of this approach. The part that is most confusing though is the appearance that the MAD flittering threshold is fixed (2) and not learnt from the data. This is a weakness as presumably they number of cells that ought to be removed in a given cluster is a function of the overall composition of cells within that cluster. The method would be improved by using an algorithm to determine the MAD threshold within a given cluster.

4.	What is the rationale for incorporating a filtering step based on fixed thresholds to determine empty drops? This seems counter intuitive given the case made for not using fixed filters for other QC metrics. Presumably there will be biological scenarios where true empty droplets remain, and cells removed. Can this QC be incorporated as one of the QC metrics on ddqc?

5.	The survey of QC practices across >100 papers is valuable, although this section is long, highly descriptive and it's not clear if this much space is required. To support the paper's narrative, I would suggest shortening and/or moving sections to supporting material. Similarly, figures 1 and 2 are repetitive and I don't feel that they add much value as main text figures.

Minor

6.	The term cell states and cell types appears to be used interchangeably. Suggest consistency is applied, or clearer rationale given for use of those specific terms.

7.	The manuscript would benefit for a clearer explanation of what ddqc does much earlier in the paper. What specific qc metrics does it estimate? It took a long time (well into the results) before these were first mentioned. I would suggest summarising in the abstract and end of introduction.

**Authors Response**

**Point-by-point responses to the reviewers’ comments:**

*Overview*

*We thank the Reviewers for the thoughtful comments, feedback, and enthusiasm for our work. We have addressed the Reviewers’ comments by adding new data and analyses, and a thorough substantial revision of the text. We first highlight the key aspects of the revision and then address each of the Reviewers’ comments point-by-point.*

*Key points in our revision include:*

*● Comparison of default ddqc clustering to different clustering methods. We completed a head-on-head comparison of the default ddqc clustering algorithm (Louvain) to 5 other graph- based and non graph-based algorithms, and concluded the results of Louvain are superior or comparable (Supplementary Text, Supplementary Figure 4A,B, Supplementary Table 4).*

*● Updates to code to expand user defined arguments. Motivated by reviewer suggestions, we have updated the code to allow flexibility for the user to define (1) hyperparameters for different steps (PCA, clustering, number of neighbors for K-NN), (2) clustering algorithms, (3) thresholds for filtering.*

*● Additional analysis to improve interpretation of results: We have added visualizations and supplementary tables to supplement the text description of the comparison between ddqc and miQC.*

*● New sensitivity analysis of clustering parameters on ddqc results. We did an extended analysis to check the impact of varying clustering hyperparameters on ddqc. We concluded that the results were robust to changing hyperparameters within the range tested (Supplementary Text, Supplementary Figure 4C,D).*

**Response to Reviewer 1**

The authors showed that QC metrics in scRNA-seq data vary with biological factors. Based on their findings, they propose ddqc, a data-driven QC filtering method. They found that ddqc can find cell-type clusters missed by the conventional data-agnostic method. The effectiveness of ddqc has been validated using many data sets, and the manuscript is expected to be beneficial to the single-cell biology community

*We thank the Reviewer for their appreciation of our work, and the value to the single-cell biology community.*

1. In the graph-based clustering step, if a cluster of low-quality cells is generated, such cells may be left behind. Is it possible to evaluate the accuracy of this point more quantitatively by simulated data?

*We appreciate the Reviewer for the question. As we described in the Discussion section, by virtue of the data-driven approach, ddqc may retain a cluster of cells which may be technical artifacts. This happens because ddqc performs QC on every individual cluster, and if a cluster is entirely technical, it becomes hard to “automatically” evaluate it as such. A lack of ground truth data for quality control assessment means that we cannot evaluate this point very quantitatively. Further, most existing simulators[1] were made for assessing biological signals or experiment design[2], and lack data properties essential for assessing QC.*

*However, sometimes, a technical cluster may also inform a particular aspect of the study (tissue degradation, stress, pathology etc) which may be useful for the study conclusions. Hence, we strongly encourage the analyst to use exploratory data analysis to determine if a cluster is entirely technical or not.* ***To facilitate the process, we return several metrics for each cell*** *(e.g. the cluster it belongs to, QC filters for each metric for the cluster etc) so that the analyst can make an informed decision. These metrics are defined in the tutorial in the “Outputs” section:* [*https://github.com/ayshwaryas/ddqc/blob/master/tutorials/ddqc_tutorial.ipynb*](https://github.com/ayshwaryas/ddqc/blob/master/tutorials/ddqc_tutorial.ipynb)

2. There are a number of hyperparameters in ddqc (e.g., number of principal components, the K and the resolution in the graph-based clustering steps). Can these values be determined independently of the underlying biological variation? It would be great if the author discuss the effect of the choice of these hyperparameters on the results.

*We thank the reviewer for raising this excellent point. ddqc uses clustering as an approach to broadly classify cells into biologically similar neighborhoods, and then applies adaptive QC on these neighborhoods, thus improving on standard quality control approaches. As the primary goal of ddqc is not to identify and annotate clusters of relevance in a given study, but rather to improve on standard quality control approaches, we initially benchmarked these parameters. In our benchmark datasets spanning 6,228,212 cells, we found that n=50 principal components, k=20 nearest neighbors, and a louvain clustering resolution of 1.4 (Seurat resolution of 1), leads to identification of expected biologically relevant cell types in the respective studies, with additional cell types recovered by ddqc.*

*Having said that, we do acknowledge that changing these hyperparameters may influence clustering.* ***To address the reviewer’s point, we have performed a benchmark of varying hyperparameters*** *for 12 combinations on the Tabula Muris (1) Heart and Aorta and (2) lung datasets as a case study. The combinations are as below:*

*Number of PCs, Number of neighbors, Clustering resolution*

*Default: 50, 20, 1.4*

*changing only resolution:*

*50,20,1*

*50,20,0.5*

*changing k and resolution:*

*50,10,1.4*

*50,10,1*

*50,10,0.5*

*changing pcs and resolution:*

*20,20,1.4*

*20,20,1*

*20,20,0.5*

*changing pcs, k, and resolution:*

*20,10,1.4*

*20,10,1*

*20,10,0.5*

*We find that the results from our default options remain robust. We have summarized the results in* ***Supplementary figure panels S4C and S5D, the Supplementary Text and Methods sections.***

*The impact of clustering parameters on cluster robustness is an actively studied field [3,4]. In general, increasing the number of principal components and clustering resolution increases the granularity of clusters and helps identify rare cells whereas a lower resolution results in broader cell classes. So to enable the user to specify their desired resolution, ddqc has the cluster resolution as a user adjustable parameter. We have now further updated the code to allow the user to specify the number of principal components and the number of neighbors. In the Python implementation of ddqc, the user is now able to adjust n_components and K parameters for the ddqc_metrics function. In the R implementation we have added similar functionality by including n.pcs and k.param parameters for the ddqc.metrics function.*

3. For Fig. 1 and 2, please specify in the figure legend how the QC metrics were aggregated (average, median, etc.) across cells in "each" study to derive the distribution "across" studies.

*We thank the reviewer for raising the detail. As indicated in the legend, each row in a panel is the distribution of QC metrics per cell in the specific tissue (Fig 1) or cell type (Fig 2) of interest within a given study. The metrics are not aggregated across studies as the goal is to show that the distribution of QC metrics varies with biology (tissue and cell type) and technical attributes (study, technology etc).*

4. Please write in the legend of Fig. 2 what the numbers in the y axis label mean.

*We thank the reviewer for pointing out this oversight. The Y-axis tick labels are the cluster numbers followed by the annotated cell type.* ***We have updated the legend*** *to clarify this information.*

5. [p. 12 line 4] The results of the comparison with minQC should be presented not only as sentences but also as a Supplementary Figure or Supplementary Table..

*We thank the reviewer for the suggestion.* ***We have supplemented the text summary of the comparison with two supplementary figure panels (Supplementary Figure 5E, F)*** *to illustrate the comparison between ddqc and miQC. Briefly, Fig S5E and S5FB are UMAP visualizations of the cells (barcodes) retained by ddqc in the Tabula muris heart and Olfactory Epithelium datasets respectively, and annotated by predicted cell type for each cluster. The green color points indicate the cells retained exclusively by ddqc and the black color points indicate the cells exclusively retained by miQC. Finally the yellow-orange color indicates cells retained by both methods.*

6. Regarding Code Availability, it would be appreciated if the requirement for R version is stated in the README on the GitHub repository (I failed to install the R package possibly due to the old version R).

*We thank the reviewer for raising this detail.* ***We have updated our GitHub readme*** *(https://github.com/ayshwaryas/ddqc_R/blob/master/readme.md) to reflect the R version (>= 4.0.0) required to appropriately install ddqc.*

**Response to Reviewer 2**

Subramanian et al. present an approach which employs a dynamic 'data driven' solution for determining QC cutoffs for single cell data. The paper describes a significant challenge in single cell analysis, and one that is often ignored or unrealised. It remains normal to apply 'commonly used' QC thresholds irrespective of the type of cells or taking into account other biologically relevant information. Indeed, the field seems to have almost accepted this, with deviations from 'common' thresholds challenged. The consequence is that many studies likely introduce some sort of bias, either though exclusion of cells / data that is meaningful or inclusion of background noise. Separating the two and dynamically drawing the correct line is challenging. The introduction is particularly good at outlining the issues, and providing some specific but well understood biological examples that underscore the necessity to develop better methods

*We thank the Reviewer for their interest and appreciation of our work and the importance of our findings, as well as for their thoughtful comments which we address below and in our revised manuscript.*

1) The argument is made that data must be partitioned by biology and QC performed on those partitions. The concern that I have is that this partitioning rests completely on clustering (graph- based), with all cells 'clustering' together then arbitrarily considered as the same and QC applied to each cluster separately. We already know that clustering is imperfect, with different cell types clustering together, and different patterns of 'noise' imitating real cell types and clustering together. My interpretation is that the problem ddqc is aiming to solve for a 'single sample' are the same for a subset of cells in a sample. Moving one level down the resolution helps somewhat, but still doesn't solve the underlying issue completely. To help demonstrate utility, it would be valuable to show performance on clusters, where cells are also independently classified, and thus your ability to show removal of poor QC cells and retention of real cells. PBMCs would be useful given the ability to accurately classify cells, and identify cells/droplets that do not align to a reference cell type. The latter are hopefully filtered by ddqc and the former kept. Showing these results would be valuable.

*Thanks for the suggestion. Similar motivations had led us to compare author provided annotations on the Tabula muris lung dataset as a case study, where we compared differences in lung cell taxonomies using ddqc and author-defined cutoffs (Figure 3D). In that particular case, ddqc did not retain additional cells which looked like poor quality.*

***Further on the reviewer’s suggestion****, we downloaded the PBMC dataset used in the Seurat tutorial and ran the tutorial (including the QC) to annotate the cells. We then ran ddqc on the dataset. On comparing, the ddqc results contained a cluster for each of the 9 cell types identified in the tutorial (see below table), where the cells of the given cell type compose more than 70% of the cluster (based on annotations from running the Seurat tutorial). No cells were retained in Seurat that were not retained by ddqc.*

2) It would also be helpful to demonstrate robustness and similar results across multiple clustering methods. This could easily be incorporated into the package.

*We thank the Reviewer for their suggestion.*

***Following the Reviewer’s comment, we compared the performance of ddqc across six clustering algorithms*** *on two case study datasets: (1) the Tabula Muris Heart and Aorta tissue and (2) the Tabula Muris lung tissue dataset as a benchmark: (1) louvain, (2) leiden, (3) spectral_louvain, (4) spectral_leiden, (5) k-means clustering, (6) hierarchical clustering, where algorithms 1-4 are provided by Pegasus library, algorithms 5 and 6 implementations is described in the Methods section. For all algorithms, default parameters were used.*

*In this head-to-head comparison, we show that all algorithms produced nearly identical results* ***(Supp. Table S4, Supp Figure 4A,B)*** *on the two case study datasets. We have compared the barcodes of cells retained by different algorithms. In the heart and aorta dataset, 2568 recovered cell barcodes post ddqc are shared among all clustering algorithms, with the final number of cells ranging from 2571(hierarchical) to 2587 (Leiden). Similar results are observed in Lung, where 15601 barcodes are shared, with the final number of barcodes ranging from 15682 (k-means) to 15892 (Leiden). In the heart, Louvain (retaining 2586 cells) performed on par with Leiden and better than other clustering methods. In lung, Leiden was the only cluster which retained more cells (40 additional pericyte cells) which were removed in the default Louvain clustering method (based on the QC metric of number of UMI counts ). We visualize the results of the comparison in Supplementary Figure panels 4A and 4B, enumerate all results in* ***Supp Table S4*** *and have updated the* ***Supplementary Text and Methods*** *section.*

*Additionally,* ***in line with the reviewer’s excellent suggestion, we have made the clustering method a user-adjustable argument*** *in the Python package for these four options which performed in general better than k-means or hierarchical (1) louvain, (2) leiden, (3) spectral_louvain, (4) spectral_leiden, and updated the tutorial to reflect these changes*

3) At a simplistic level, ddqc appears to apply MAD on a cluster on a single parameter (QC metric). It would help to make a stronger case for the novelty of this approach. The part that is most confusing though is the appearance that the MAD flittering threshold is fixed (2) and not learnt from the data. This is a weakness as presumably they number of cells that ought to be removed in a given cluster is a function of the overall composition of cells within that cluster. The method would be improved by using an algorithm to determine the MAD threshold within a given cluster.

*We thank the Reviewer for this suggestion and agree that a data-driven MAD threshold might indeed provide new insights. However, modifying the QC parameter would substantially change the current method ddqc on the whole, warranting a new set of analysis and possibly a follow up manuscript. Given that our manuscript is already lengthy with diverse analyses making a case for data-driven QC, and that a parameter such as MAD that depends on the median of a data cloud is data-driven to some extent, we believe that this method's improvement is beyond the scope of the current manuscript and should be addressed in future manuscripts.*

4) What is the rationale for incorporating a filtering step based on fixed thresholds to determine empty drops? This seems counterintuitive given the case made for not using fixed filters for other QC metrics. Presumably there will be biological scenarios where true empty droplets remain, and cells removed. Can this QC be incorporated as one of the QC metrics on ddqc?

*Thanks for the question. By default, the ddqc software allows these preprocessing filters to be user-tunable: the user is able to specify the lower filter for number of genes (ngenes) and upper filter for the percentage of reads mapping to mitochondrial genes (percent mito).*

*Our initial logic for including these filters in our analysis is the lack of computational power in current scRNA-seq analysis pipelines to complete analysis of all the raw barcodes emerging from a droplet-based single cell transcriptomic profiling technology such as 10x. For most of the datasets analyzed in our manuscript, not removing cells with very low gene complexity (empty droplets) leads to an input on the order of millions of cells causing current scRNAseq analysis algorithms and workflows to fail at the clustering step for most datasets if we don’t place a filter. On average, we found a filter of 100 genes to work well across both small and large datasets. For e.g. even in the Tabula Muris heart and aorta dataset which is relatively small (starting number of cells: 2722), the clustering step fails if we attempt to reduce this threshold to ngenes < 50 whereas for larger datasets (e.g.[5]), the pipeline fails for ngenes < 100.*

5) The survey of QC practices across >100 papers is valuable, although this section is long, highly descriptive and it's not clear if this much space is required. To support the paper's narrative, I would suggest shortening and/or moving sections to supporting material. Similarly, figures 1 and 2 are repetitive and I don't feel that they add much value as main text figures.

*We thank the Reviewer for this suggestion. We have now abbreviated the main text section of the QC survey and created a separate supplemental text section which goes into the details.*

Minor:

6) The term cell states and cell types appears to be used interchangeably. Suggest consistency is applied, or clearer rationale given for use of those specific terms.

*We thank the Reviewer for the apt remark. We have amended the text to reflect the distinction between cell type and cell state (line nos 85-86, 167, 234-236) and remained consistent with the use of “cell-type” as that has been the major emphasis in the manuscript.*

7) The manuscript would benefit for a clearer explanation of what ddqc does much earlier in the paper. What specific qc metrics does it estimate? It took a long time (well into the results) before these were first mentioned. I would suggest summarizing in the abstract and end of introduction.

*We thank the reviewer for the suggestion. We have now incorporated a summary of ddqc in the abstract (line no 37-39 ) and at the end of the introduction (line no 112-114 ) as suggested by the reviewer.*

*References*

*1. Cao Y, Yang P, Yang JYH. A benchmark study of simulation methods for single-cell RNA sequencing data. Nat Commun. 2021;12:6911.*

*2. Li WV, Li JJ. A statistical simulator scDesign for rational scRNA-seq experimental design. Bioinformatics. 2019;35:i41–50.*

*3. Patterson-Cross RB, Levine AJ, Menon V. Selecting single cell clustering parameter values using subsampling-based robustness metrics. BMC Bioinformatics. 2021;22:39.*

*4. Krzak M, Raykov Y, Boukouvalas A, Cutillo L, Angelini C. Benchmark and Parameter Sensitivity Analysis of Single-Cell RNA Sequencing Clustering Methods. Front Genet. 2019;10:1253.*

*5. Tucker NR, Chaffin M, Fleming SJ, Hall AW, Parsons VA, Bedi KC Jr, et al. Transcriptional and Cellular Diversity of the Human Heart. Circulation. 2020;142:466–82.*

**Second round of review**

**Reviewer 1**

The authors sufficiently responded to all of the reviewers' comments.

**Reviewer 2**

1. In my original review I suggested that to help demonstrate utility, it would be valuable to show performance on data where cells are also independently classified, and thus your ability to show removal of poor QC cells and retention of real cells. This was based on my interpretation is that the problem ddqc is aiming to solve for a 'single sample' are the same for a subset of cells in a sample. Moving one level down the resolution helps somewhat, but still doesn't solve the underlying issue completely. To confirm this, it's critical the ddqc is performed on data where the cells have been independently classified, rather than clustered and then labelled together. The reason is that we anticipate clustering to be inaccurate for some cells, and so a ground truth is hard to identify. In addressing this, both examples where ddqc was applied involve cell classification on clustered cells, not independently classified cells. For the reasons stated above, I believe it is important to independently classify cell data. A suggestion would be to use the 10x PBMC data and re-classify using an independent cell classification method, then apply ddqc on annotated cells, rather than relying on the original cluster labels from 10x.

2. One option to dynamically estimate MAD for a given dataset, would be to iteratively change the MAD, calculate the number of droplets removed, and then 'decide' on a MAD based on the asymptote of the number of droplets removed.

**Authors Response**

**Point-by-point responses to the reviewers’ comments:**

**Response to Reviewer 1**

The authors sufficiently responded to all of the reviewers' comments.

*We are thrilled that reviewer 1 found our first round of revisions satisfactory and has no further*

*comments or concerns.*

**Response to Reviewer 2**

1. In my original review I suggested that to help demonstrate utility, it would be valuable to show performance on data where cells are also independently classified, and thus your ability to show removal of poor QC cells and retention of real cells. This was based on my interpretation is that the problem ddqc is aiming to solve for a 'single sample' are the same for a subset of cells in a sample. Moving one level down the resolution helps somewhat, but still doesn't solve the underlying issue completely. To confirm this, it's critical the ddqc is performed on data where the cells have been independently classified, rather than clustered and then labelled together. The reason is that we anticipate clustering to be inaccurate for some cells, and so a ground truth is hard to identify. In addressing this, both examples where ddqc was applied involve cell classification on clustered cells, not independently classified cells. For the reasons stated above, I believe it is important to independently classify cell data. A suggestion would be to use the 10x PBMC data and re-classify using an independent cell classification method, then apply ddqc on annotated cells, rather than relying on the original cluster labels from 10x.

*We thank the reviewer for clarifying the comment and we regret that our first revision did not adequately address it. To thoroughly address the comment, we independently annotated cells of the PBMC dataset using three different automated annotation methods including both classification and non-classification based strategies:*

*1) SingleR (https://bioconductor.org/packages/release/bioc/html/SingleR.html) [1]*

*2) Azimuth (https://azimuth.hubmapconsortium.org) [2]*

*3) CellTypist (https://www.celltypist.org/) [3]*

*Each of these methods assigns a cell type label to each cell (We note that neither of these independent approaches throws out any cells as “unannotable” regardless of quality suggesting some inherent bias/inaccuracy in the approaches themselves.). Thus, we had three independent annotations for each cell (by each of these methods). In contrast, ddqc uses clustering to dissect biological heterogeneity and group cells into clusters, representing their approximate biological celltype. We looked at the overlap of the ddqc cluster IDs with independent annotations and note that ddqc cluster IDs had high one-one correspondence to independent annotation groups (Please see Appendix I below).*

*ddqc applies MAD-based QC on clusters. To compare the results of independent classification with that from ddqc, we grouped cells with the same automated cell-type annotation and applied MAD-based filtering on each such group, independently for each of the methods.* ***Table S5*** *provides a mapping between ddqc results post clustering and post each of the independent classification methods. A comparison of cells removed by MAD-based filtering revealed no* ***differences*** *between independent classification and clustering based approaches. In all cases, the same 6 cells were filtered out highlighting that clustering performs on par with other independent methods for the purposes of ddqc.*

*To compare a second independent example, we compared the human lung dataset [4] which has served as a use case in* ***Figure S5*** *of the manuscript. Azimuth unfortunately could not handle cells even from a single patient sample (please see screenshot below in Appendix II) because it was too large; and hence, we only use CellTypist for this comparison.* ***Table S5*** *provides a mapping between ddqc results after clustering and each of the independent classification methods.*

*We note that both methods retain 115,591 cells comprising 98.9% of ddqc results and 99.9% of CellTypist results, showing high concordance.*

*Summary of results:*

*Total number of cells in Krasnow Lung after initial QC (> 100 genes, < 80% mito): 122,301*

*Cells that are retained by ddqc post clustering: 116,854*

*Cells that are retained by ddqc post CellTypist: 115,672*

*Intersection of ddqc post clustering and post CellTypist: 115,591 (98.9% of ddqc results; 99.9% of CellTypist results)*

*Cells that are uniquely retained by ddqc post clustering: 1263 (1% of input)*

*Cells that are unique retained by ddqc post CellTypist: 81 (0.06% of input)*

*We attempted to apply independent classification methods to datasets besides the PBMC and lung examples, however, all three approaches (SingleR, Azimuth, CellTypist) are currently limited in the range of tissues they can annotate and only provide capabilities for ~5 example organs, mostly human data. Otherwise, one must provide a well-annotated training or reference dataset. Based on our comparisons of PBMC and human lung datasets, and in consideration of the above limitations, we conclude that clustering is a reasonable method to use until reference atlases for multiple tissues are curated by the community and independent classification methods become routine and well tested which we anticipate will take a few years.*

*Appendix 1: Table of comparison of ddqc cluster IDs with automated cell type labels*

*SingleR:*

*Azimuth:*

*CellTypist:*

*Appendix II: Azimuth run issues “Too many cells. Try another dataset” error*

*Test sample 1: P1_1*

*Test sample 2: P2_1*

2. One option to dynamically estimate MAD for a given dataset, would be to iteratively change the MAD, calculate the number of droplets removed, and then 'decide' on a MAD based on the asymptote of the number of droplets removed.

“The reason for suggesting a learnt MAD threshold and incorporating thresholds from empty droplets / damaged cell identification methods is to ensure that their method is ‘biology inspired’ – i.e. data-driven. My thinking is aligned to the authors, in that in a given single-cell data set, there will be significant biological variation in ‘true’ cells around the MAD and damaged cell thresholds (this has been shown for damaged cells – “Muskovic, Walter, and Joseph E. Powell. "DropletQC: improved identification of empty droplets and damaged cells in single-cell RNA-seq data." Genome biology 22.1 (2021): 1-9”), such that dataset to dataset, there will be more or fewer actual cells either side of a fixed MAD threshold. There is no one threshold will work equally well for all datasets. As such, learning a MAD threshold from the data and applying that should improve the performance of their method. Including damaged cells, thresholds will also have a considerable effect as there is enormous variation between datasets, and these methods are robust now.

I also believe this suggestion aligns with the philosophy of their approach. At the moment, it is a bit hard to understand the justification for using a data-driven approach that starts with fixed filtering that will vary the proportion of actual cells removed and false cells kept from one data set to another. As such, I think it is important to include an approach for learning MAD and incorporating damaged cell thresholds.”

*We thank the reviewer for their thoughtful comments and recommendations and we have attempted to thoroughly address the suggestion via several approaches. We first recall that cell QC broadly takes place at four levels:*

*1) Ambient or empty droplet removal where tools such as EmptyDrops [5], CellBender [6] and DropletQC [7] are used.*

*2) Damaged cell removal as defined in DropletQC as those with low UMI and high “nuclear fraction.” It remains to be widely-tested if such cells can provide biologically useful information.*

*3)* ***QC of cells based on gene complexity and %mito, %ribo etc which has been arbitrary in the field thus far and defines the scope of the current manuscript ddqc.***

*4) Doublet removal where several tools such as DoubletFinder [8], scrublet [9], DoubletDetector [10] have been developed and benchmarked[11] .*

*ddqc specifically addresses the third case since there are existing methods for the other approaches as the reviewer also notes. To address the comment, we break down our response into two parts:*

***PART A: The reviewer raises a concern of applying “fixed” basic filtering.***

*(1) Considering the reviewer’s recommendations on existing methods that remove empty drops in a data-driven manner, we have now tested two additional methods to remove empty droplets and evaluated its impact on ddqc.*

*A. First, we applied EmptyDrops [5] using the R package DropletUtils [12] on two of our datasets (Tabula muris Lung and Heart and Aorta) to remove empty droplets and then use the called “cells” as input to ddqc. We note that depending on the dataset, the default initial filters (“default”) in ddqc can be more conservative or more liberal (Appendix III) in comparison to EmptyDrops. However, on applying ddqc downstream on cells retained by both approaches, there was high concordance regardless of the basic filtering approach used suggesting that the approach is quite robust to empty droplet filtering methods:*

*Lung Tabula muris*

*Number of cells retained after both default and emptydrops: 12652*

*Total cells default after ddqc: 12477*

*Total cells emptydrops after ddqc: 12459*

*Cells in common: 12412*

*Cells unique to default: 65*

*Cells unique to emptydrops: 47*

*Heart Tabula muris*

*Number of cells retained after both IQC and emptydrops: 2676*

*Total cells IQC after ddqc: 2565*

*Total cells emptydrops after ddqc: 2572*

*Cells in common: 2562*

*Cells unique to IQC: 3*

*Cells unique to emptydrops: 10*

*B. Second, we applied DropletQC https://powellgenomicslab.github.io/DropletQC/articles/DropletQC.html which was published during our revision process, and works directly with BAM and BAI files. DropletQC works on two dependent steps: first it identifies empty droplets, and then post annotation, identifies damaged cells. There were two issues with DropletQC. For both our test TM datasets, the empty droplet identifier identify_empty_drops function did not work because of the large input size. On requesting the authors (please see GitHub PR: https://github.com/powellgenomicslab/DropletQC/issues/3), the author’s kind recommendation was to apply a fixed cutoff of 1000 UMIs before running DropletQC (their definition of what a cell is). However, doing this would defeat the purpose of not using arbitrary fixed cutoffs as the reviewer raises. So we did not proceed further as all downstream steps depend on these initial results, especially when computationally robust alternatives (such as both our initial filtering as well as EmptyDrops) are available.*

*C.* ***On a related note, the reviewer suggests using “damaged cells,”*** *a useful concept that has been described in the paper DropletQC. For actually identifying damaged cells, DropletQC would require us to first identify empty droplets by using their inherent identify_empty_drops which as described above in A.1(b), required us to impose cutoffs. In addition, damaged cell identification requires cell annotation first. So we did not proceed with it as these steps necessitate arbitrary filters. Interested users may apply it following ddqc.*

*(2) We agree with the reviewer that applying ddqc directly on the complete input datasets would have been ideal, however, our rationale for using these basic filters was simply that it was not computationally efficient or feasible to include the full dataset in existing dimensionality reduction and clustering implementation pipelines. By default, the ddqc software allows these preprocessing filters to be user-tunable: the user is able to specify the lower filter for number of genes (ngenes) and upper filter for the percentage of reads mapping to mitochondrial genes (percent mito). On average, we found a filter of 100 genes to work well across both small and large datasets. For e.g. even in the Tabula Muris heart and aorta dataset which is relatively small (starting number of cells: 2722), the clustering step fails if we attempt to reduce this threshold to ngenes < 50 whereas for larger datasets (e.g.[13]), the pipeline fails for ngenes < 100. We set these manual thresholds that are very conservative. The main purpose is to reduce the size of the data. This minimal filtering helps a lot with downstream steps. We agree that these cutoffs can be arbitrary and have hence updated our code and documentation to enable the user to turn off the default filtering and supply (1) filtered Cellranger matrices which applies Emptydrops by default or (2) apply Emptydrops or their cell caller of choice and provide preprocessed files or (3) provide their own filters (as opposed to the default) extending it to all three QC metrics. In conclusion, ddqc works well regardless of whether Emptydrops (DropletUtils) or the basic filtering cutoff is used, and is amenable to user-defined parameters or processed input files.*

***PART B: Using a data-driven MAD:***

*As the reviewer suggested, we varied the MAD parameter for multiple datasets for which results may be found as the supplementary reviewer file* ***PBP_reviewerfiles_adaptivemadthreshold.zip.*** *We iterated through the MAD multiplier (1-3.5) in increments of 0.1, computing the number & % of cells removed at each iteration. Based on these results we produced several plots:*

*● The density distribution of each QC metric using each cluster (using the R library ggridges joyplot with rug broken down by cluster for each QC metric). Lines representing 1 * MAD (red), 2 * MAD (green), and 3 * MAD (blue) for each cluster were added to these plots in red, green, and blue colors respectively (****PBP Figure 1,2****).*

*● Linechart with number or percentage of cells filtered on y-axis and threshold on x-asis faceted by cluster. (this plot was also included in the released version of pegasus implementation of ddqc) (****PBP Figure 3****).*

*We observed that the results are relatively robust and the general conclusions hold across datasets. We discuss results on two case studies here: the Tabula muris heart and Human adipose datasets.*

*(1) To evaluate if different clusters within a dataset vary in the spread and distributions of their QC metrics, we first confirmed that the density distribution of the QC metrics were largely unimodal by running the following functions:*

*● dip.test() from the R package diptest (*[*https://cran.r-project.org/web/packages/diptest/index.html*](https://cran.r-project.org/web/packages/diptest/index.html)*)*

***PBP Figure 1*** *: Per cluster density distributions of QC metrics*

*Human adipose tissue (left nGenes, a; right percent.mito, b):*

*Tabula muris heart (left nGenes, c; right percent.mito, d):*

*● is.unimodal() from the R package LaplacesDemon (*[*https://cran.r-project.org/web/packages/LaplacesDemon/index.html*](https://cran.r-project.org/web/packages/LaplacesDemon/index.html)*))*

*For each cluster we looked at n_counts, n_genes, and percent_mito and assessed whether it was unimodal. We considered the cluster as unimodal if p_value was less than 0.05 for diptest and if is.unimodal returned TRUE for LaplacesDemon. We observed that the majority of the clusters (****PBP Figure 1****) in our test datasets were unimodal (nUMI: 79.4-94%, nGene: 73-89.25%, percent.mito: 76-83%) validating an approach that filters off the extreme tail (over a mixture model assuming explicit number of modes). In earlier iterations of ddqc, we had found MAD to be more robust and data-driven than using percentile approaches which may be too strict or statistical outliers (Tukey’s fences) which often tend to be very liberal.*

***PBP Figure 2****: Per cluster histogram of MAD. Human adipose tissue:*

*Tabula muris heart:*

*2) We recorded other information, such as the cluster’s median, MAD, standard deviation, and MAD to SD ratio for n_counts, n_genes, and percent_mito (PBP Figure 2). The MAD varied from cluster to cluster and in the majority of cases, the ratio of MAD to standard deviation was < 1.*

*(3) We next observe the trend of the number of cells filtered as we sweep along the MAD multiplier (PBP Figure 3). Specifically, we note three trends in the line charts which vary cluster to cluster:*

*1. Early single knee point*

*2. multiple knee points,*

*3. Slope or gradient*

*Tabula muris heart:*

*1. Early single-knee: In clusters 1-3, 5-7, 10-11, 14-15, 18, the number of cells lost quickly drops to a constant in the multiplier range of 1-1.2 and results remain robust for the multiplier=2.*

*2. multiple knee points: Clusters 9,13,17: there are multiple inflection points and it is a bit unclear where to draw the “optimal” cutoff. In most cases, x=2 is midway. In rare cases, we had a single knee.*

*3. slope or gradient: Cluster 4*

*Human Adipose:*

*1. Early single-knee: In clusters 1,9,15,17,19-20, the number of cells lost quickly drops to a constant in the multiplier range of 1-1.2 and results remain robust for the multiplier=2.*

*2. multiple knee points: Clusters 4,21: there are multiple inflection points and it is a bit unclear where to draw the “optimal” cutoff. In most cases, x=2 is midway. In rare cases, we had a single knee.*

*3. slope or gradient: Cluster 11,13*

*In cases (2) and (3), we need a cost function to optimize the appropriate cutoff and such a cost function is hard to determine in advance because of the wide variance between datasets, cohorts and cell types, and their associated tradeoffs. A MAD multiplier of 2 is quite robust in all three cases across the datasets we examined.*

***PBP Figure 3: Per cluster trends in number of cells filtered out (y-axis) when sweeping the MAD multiplier (x-axis) in increments of 0.1.***

*Human adipose tissue:*

*Mouse and heart aorta:*

*Based on our case studies, we think the best approach would be to empower the analyst to make such decisions depending on their study design. We have updated our code to provide the sweep plots and the input argument to specify the MAD multiplier for each metric thus increasing the flexibility and adapting to specific data characteristics while keeping multiplier=2 as defaults. In addition to MAD, the user can also choose to pick statistical outliers (based on Tukey’s fences). While automating the process is becoming a recognised problem in the field, the idea of applying QC to each cell type is in itself not widely adopted and what we hope ddqc can encourage.*

*In summary, based on our extensive testing, we have made changes to ddqc to provide the user with (1) interactive analyses and (2) options to specify the MAD cutoffs for each cluster and vary results. We appreciate the reviewer for encouraging us to consider this with rigor and we hope this will improve the QC experience for the community.*

*Appendix III*

*References:*

*1. Aran D, Looney AP, Liu L, Wu E, Fong V, Hsu A, et al. Reference-based analysis of lung single-cell sequencing reveals a transitional profibrotic macrophage. Nat Immunol. 2019;20:163–72.*

*2. Hao Y, Hao S, Andersen-Nissen E, Mauck WM 3rd, Zheng S, Butler A, et al. Integrated analysis of multimodal single-cell data. Cell. 2021;184:3573–87.e29.*

*3. Domínguez Conde C, Xu C, Jarvis LB, Rainbow DB, Wells SB, Gomes T, et al. Cross-tissue immune cell analysis reveals tissue-specific features in humans. Science. 2022;376:eabl5197.*

*4. Travaglini KJ, Nabhan AN, Penland L, Sinha R, Gillich A, Sit RV, et al. A molecular cell atlas of the human lung from single-cell RNA sequencing. Nature. 2020;587:619–25.*

*5. Lun ATL, Riesenfeld S, Andrews T, Dao TP, Gomes T, participants in the 1st Human Cell Atlas Jamboree, et al. EmptyDrops: distinguishing cells from empty droplets in droplet-based single-cell RNA sequencing data. Genome Biol. 2019;20:63.*

*6. Fleming SJ, Marioni JC, Babadi M. CellBender remove-background: a deep generative model for unsupervised removal of background noise from scRNA-seq datasets [Internet]. Cold Spring Harbor Laboratory. 2019 [cited 2021 Mar 4]. p. 791699. Available from: https://www.biorxiv.org/content/10.1101/791699v1.abstract*

*7. Muskovic W, Powell JE. DropletQC: improved identification of empty droplets and damaged cells in single-cell RNA-seq data. Genome Biol. 2021;22:329.*

*8. McGinnis CS, Murrow LM, Gartner ZJ. DoubletFinder: Doublet Detection in Single-Cell RNA Sequencing Data Using Artificial Nearest Neighbors. Cell Syst. 2019;8:329–37.e4.*

*9. Wolock SL, Lopez R, Klein AM. Scrublet: Computational Identification of Cell Doublets in Single-Cell Transcriptomic Data. Cell Syst. 2019;8:281–91.e9.*

*10. Shor, Gayoso. DoubletDetection. GitHub repository.*

*11. Xi NM, Li JJ. Benchmarking Computational Doublet-Detection Methods for Single-Cell RNA Sequencing Data. Cell Syst. 2021;12:176–94.e6.*

*12. Lun, Griffiths, McCarthy. DropletUtils: utilities for handling single-cell droplet data. R package version 099.*

*13. Tucker NR, Chaffin M, Fleming SJ, Hall AW, Parsons VA, Bedi KC Jr, et al. Transcriptional and Cellular Diversity of the Human Heart. Circulation. 2020;142:466–82.*

**Third round of review**

**Reviewer 2**

I thank and appreciate the author's efforts in revising their work.
